# Development of Peptidomimetic PROTACs as Potential Degraders of 3-Chymotrypsin-like Protease of SARS-CoV-2

**DOI:** 10.3390/ijms26083903

**Published:** 2025-04-21

**Authors:** Chao Wei, Yuhua Li, Lina Guo, Zhiyu Shao, Hua Diao

**Affiliations:** 1 College of Chemistry and Chemical Engineering, Donghua University, Shanghai 201620, China; 15860797763@163.com; 2 NHC Key Lab of Reproduction Regulation, Shanghai Engineering Research Center of Reproductive Health Drug and Devices, Shanghai-MOST Key Laboratory of Health and Disease Genomics, Shanghai Institute for Biomedical and Pharmaceutical Technologies, Shanghai 200237, China; li_yuhua@163.com (Y.L.); guoln90@126.com (L.G.)

**Keywords:** 3CL protease, proteolysis-targeting chimeras, targeted protein degradation, thalidomide derivatives, lenalidomide derivatives

## Abstract

3CL protease (3CL^pro^), a key enzyme of SARS-CoV-2 replication, is one of the most selective targets of antivirals, as no homologous protease has been recognized in the human body. As proteolysis-targeting chimeras (PROTACs) are superior to traditional inhibitors, based on the reported cereblon (CRBN) ligands thalidomide and lenalidomide, 3CL^pro^ ligands of peptidomimetic inhibitors, and suitable linkers, we aimed to develop novel PROTACs that may trigger efficient intracellular 3CL^pro^ degradation through a balance of hydrophilicity and lipophilicity. In brief, we designed and synthesized 5 PROTAC molecules. The 3CL^pro^ degradation efficiency of the PROTACs was assayed in stable SARS-CoV-2 3CL^pro^ expression HEK293 cell models and evaluated by Western blot. All compounds showed prominent 3CL^pro^ degradation activity with tolerable HEK293 cytotoxicity. The most prominent PROTAC compounds, **15** and **16**, have DC_50_ values of approximately 1 µM, and D_max_ of 89.3% and 75% respectively, indicating good potential for further application.

## 1. Introduction

The 3-pancreatic trypsin-like protease (3CL^pro^) of SARS-CoV-2, also known as the main protease (M^pro^), is a key enzyme for viral proliferation [1]. It is a target for the development of anti-SARS-CoV-2 drugs [2], as it differs from other proteases in the human body, with the expectation to be targeted to effectively inhibit the spread of the virus. The 3CL protease is highly conserved among all coronaviruses, and the structures of its active sites are very similar, usually consisting of four sites (S1′, S1, S2, and S4) [3]. Therefore, its inhibitors or emerging degraders have the potential to be developed as broad-spectrum antivirals against coronaviruses.

Based on the characteristics of 3CL protease, researchers have developed a variety of 3CL protease inhibitors, including peptide and peptidomimetic inhibitors [4]. These inhibitors possess a specific electrophilic group, such as aldehyde, α-ketoamide, Michael acceptor, cyano, etc. These groups can form covalent bonds with the sulfur atom of the Cys145 residue, thereby inhibiting the activity of 3CL protease [5]. Peptide drugs have their advantages and can be used for the prevention and treatment of various infectious diseases [6]. Currently, the short peptide inhibitors with inhibitory activity against SARS-CoV-2 3CL^pro^ mainly include peptidomimetic compounds of peptide aldehyde and peptide α-ketoamide [7].

Small molecule inhibitors targeting the 3CL protease of the coronavirus have been extensively developed, and their mechanisms of action can be roughly divided into two categories: (1) peptidomimetic inhibitors; (2) non-peptidomimetic small molecule inhibitors. The former mainly transforms the substrate polypeptide sequence of the 3CL protease, and structurally, the reactive warhead can covalently bond with the cysteine in the catalytic center of 3CL^pro^, thereby inhibiting the activity of the 3CL protease, and most of them are covalent inhibitors. The latter is obtained through structural remodeling of existing protease inhibitors, virtual screening, from natural products, etc. [8].

Proteolysis-targeting chimeras (PROTACs) are emerging antivirals, relying on “event-driven” pharmacological mechanism rather than “occupancy-driven” mechanism of inhibitors [9,10,11]. The PROTAC molecule is a heterogeneous bifunctional small molecule, composed of a ligand that binds to the target protein and a ligand that binds to the E3 ligase, and the ligands are connected through a small molecule linker. Recently, proof-of-concept studies have shown remarkable efficacy of a GC-376 based PROTAC and a PROTAC MPD2 in diminishing 3CL^pro^ protein levels of SARS-CoV-2 [12,13].

After entering the cell, the PROTAC molecule simultaneously binds to the target protein and the E3 ligase, and recruits E2 ligase (through E3) to ubiquitinate the target protein. The modified target protein is then degraded into amino acids by the 26S proteasome system [14], and the released PROTAC molecule can be reused. PROTACs have an essential difference from traditional inhibitors. They can directly degrade the target protein, thereby more effectively regulating the function of the target protein than traditional inhibitors. Their control over the activity of the target protein is also superior to that of traditional inhibitors [15]. PROTACs do not rely on inhibiting the activity of proteins, but only need to bind to the POI (protein of insterest). They can neutralize any target that contains a region or pocket with sufficient affinity. This makes PROTACs particularly attractive for dealing with scaffold proteins, pseudokinases, and transcription factors [16].

In this paper, thalidomide and lenalidomide were used as CRBN ligands, and various linker chains and dipeptide compounds were connected under the condition of EDCI (1-(3-Dimethylaminopropyl)-3-ethylcarbodiimide hydrochloride) as a condensing agent. Ultimately, 5 PROTAC compounds with targeted protein degradation activity were synthesized.

## 2. Results and Discussion

Our design rationale for making PROTAC involved tagging a known binder of 3CL protein. This peptidomimetic compound is designed by mimicking the natural peptide substrate. It is mainly based on the substrate peptide sequence of the 3CL^pro^ and is mostly a non-covalent inhibitor. The active formyl or cyano groups that may undergo a nucleophilic addition reaction with the thiol group of the cysteine in the catalytic center of 3CL^pro^ to form a covalent bond were avoided to facilitate the release and reuse of potential PROTAC molecules for efficient target protein degradation. Accordingly, we designed the PROTAC molecules by connecting peptidoid compound and thalidomide (as a ligand for E3 ligase).

**Scheme 1:**
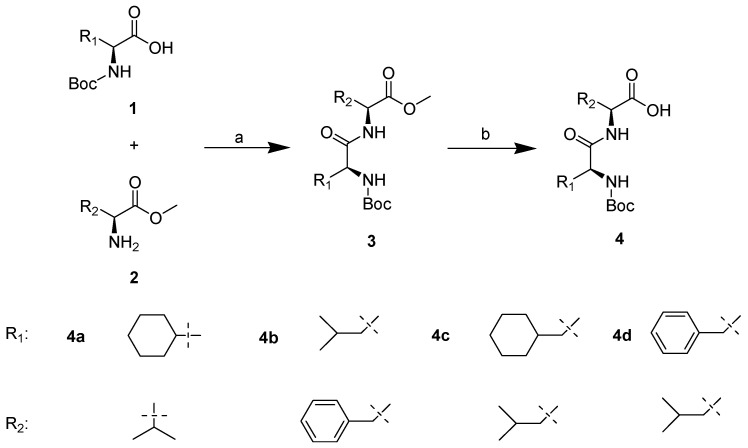
Synthesis of peptidomimetic compound (**a**) EDCI, DMAP, DIPEA, 0 °C, 8 h. (**b**) LiOH, H_2_O_2_, H_2_O, THF, 0 °C, 6 h.

**Scheme 2:**
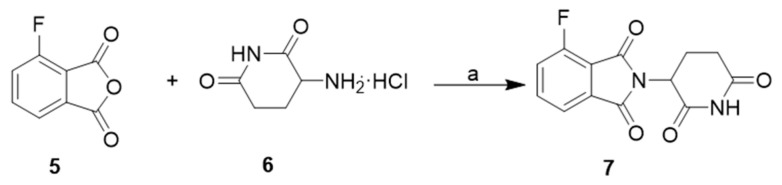
Synthesis of thalidomide derivative **7** (**a**) KOAc, HOAc, 90 °C, 8 h,75%.

We began the synthesis of the peptidomimetic compound by treating the amino acid with DMAP and EDCI in the presence of DIPEA in dichloromethane, resulting in the formation of compound **3** in good yield. The subsequent reaction with LiOH/H_2_O_2_/H_2_O in tetrahydrofuran at an ice bath yielded compound **4**, as shown in Scheme 1. The thalidomide derivative 4-flurothalidomide (7) was synthesized using the previously reported procedure [17], as illustrated in Scheme 2.

**Scheme 3:**
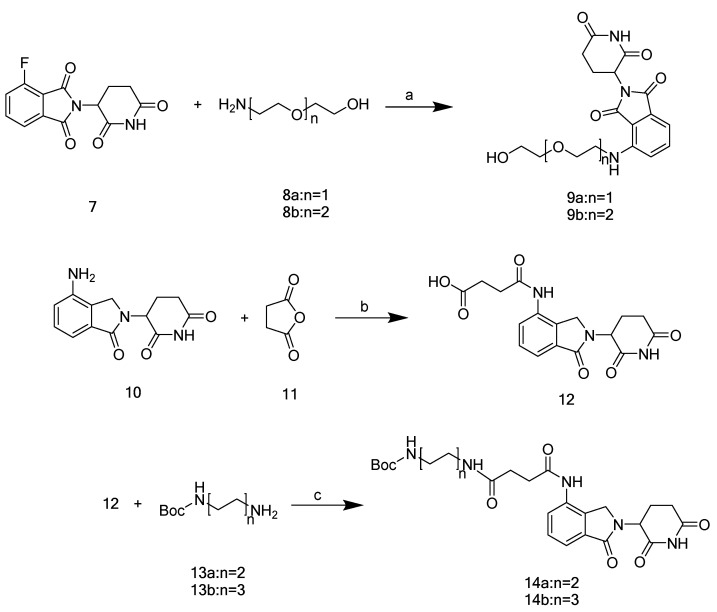
Synthesis of linkers. (**a**) DIPEA, DMF, 145 °C, 4 h, 68%. (**b**) Acetonitrile, DMF, 80 °C, 4 h, 69%. (**c**) EDCI, DMAP, DIPEA, 0 °C, 8 h, 64%.

**Scheme 4:**
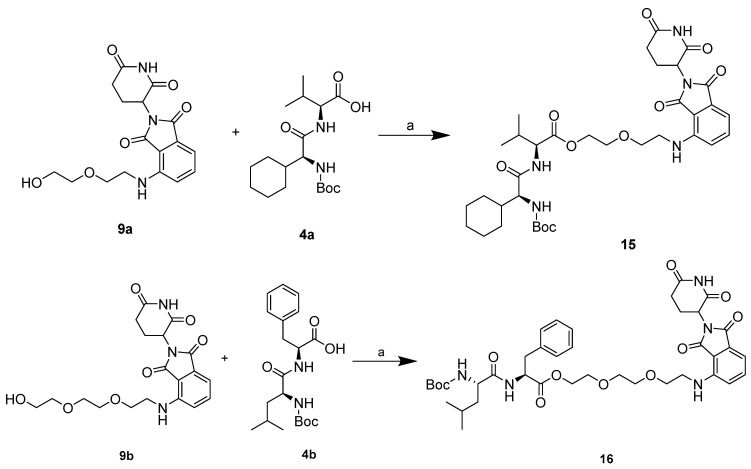

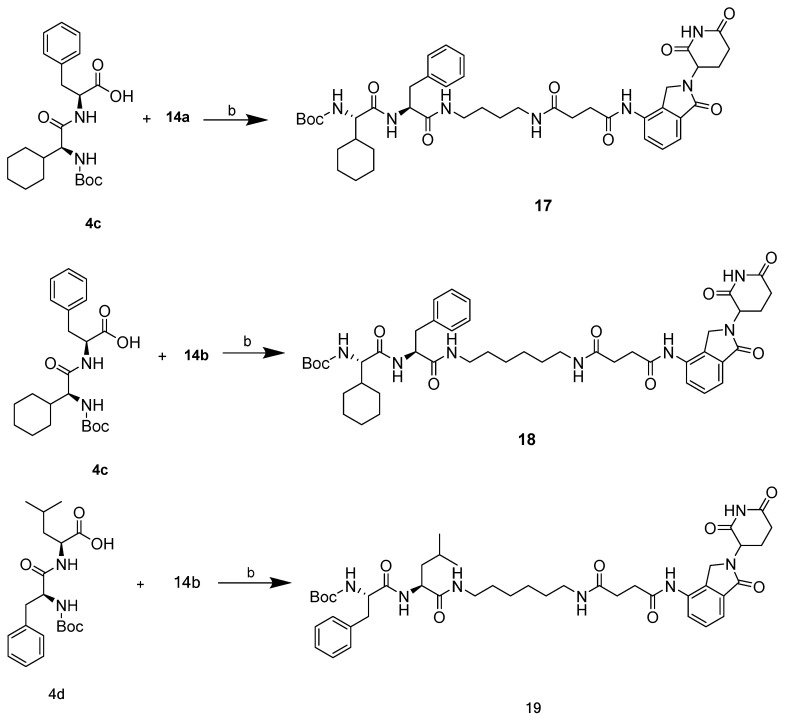
Synthesis of PROTACs **15**–**19**. (**a**) DIPEA, HOAT, EDCI, dichloromethane, 0 °C, 10 h, 37% for **15**, 31% for **16**. (**b**) Trifluoroacetic acid 2 h for **14a**/**14b**; DIPEA, diphenylhypophosphoryl chloride, DMF, 0°C, 10 h, 41% for **17**, 44% for **18**, 80% for **19**.

The linkers were connected to the thalidomide and lenalidomide, as shown in Scheme 3. Firstly, fluoro-substituted thalidomide reacted with diethylene glycol amine/triethylene glycol amine to obtain compound **9**. For the synthesis of lenalidomide derivatives, lenalidomide (purchased from Adamas-beta) reacted with succinic anhydride to obtain compound **12**. Then, **12** was condensed with mono-Boc butanediamine/hexanediamine to obtain compounds **14a** and **14b**. Then, **14a** and **14b** were stirred in trifluoroacetic acid to cleave BOC group. Finally, the PROTACs were synthesized by condensing with compound **4** in dichloromethane under the catalysis of HOAT and EDCI, as shown in Scheme 4. The copies of ^1^H and ^13^C spectra for all prepared compounds are given in Supplementary Materials. The products were also analyzed by high-resolution mass spectrometry.

**Figure 1:**
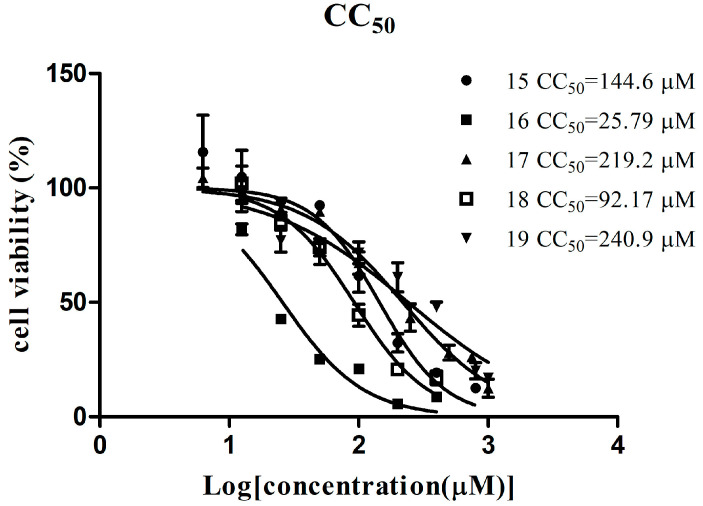
Cytotoxicity (CC_50_) of the compounds **15**–**19** is determined with HEK293. HEK293 cells were incubated with a series of increasing concentrations of compounds **15**–**19** for 72 h and cell viability was measured with cell counting kit-8 (CCK8). CC_50_: 50% cytotoxicity concentrations.

HEK293 cell clones with stable 3CL^pro^ expression were constructed after transformation with plasmid pcDNA3.1-3CL-c-flag and selected with G418. With compounds **15**–**19** in hand, we examined the dose-dependent effect of compounds **15**–**19** on the cell viability of HEK293. The preliminary investigation suggested that the cytotoxicity of compounds **15**–**19** is within acceptable ranges, as shown by the cell viability data in Figure 1. The CC_50_ values of all compounds except **16** are higher than 50 μM. Although the CC_50_ of compound **16** is approximately 25 μM, it is much higher than the DC_50_ values determined in the intracellular 3CL^pro^ degradation assays.

**Figure 2:**
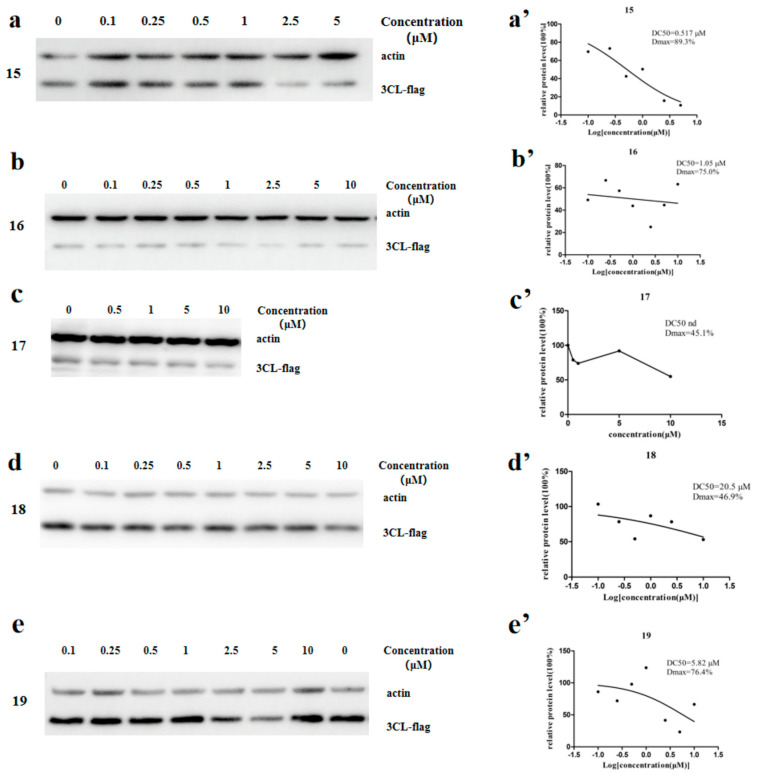
Western blot analysis of 3CL protein in HEK293 cell line. (**a**–**e**) Western blot analysis of 3CL-flag protein in the HEK293 cells with stable 3CL^pro^ expression after treatment with the indicated concentrations of **15**–**19** for 72 h. Actin was used as a loading control. (**a′**–**e′**) DC_50_ and Dmax values derived from densitometric analysis of 3CL-flag immunoblots. DC_50_: compound concentration required to degrade 50% of the target protein; D_max_: maximum degradation in percentage; nd: not determined.

We further evaluated the proteolysis effect of compounds **15**–**19** on 3CL^pro^ (Figure 2). Our Western blotting data indicated that all the compounds had obvious degradation effect on 3CL^pro^. However, despite the structural similarities among the compounds, the results differed regarding DC_50_ and D_max_ values. For some compounds, like **16**, an increase in concentration led to a decrease in degradation ability, which we speculate is due to the HOOK effect. In comparison of the degradation abilities of compounds **15–19**, compounds **15**, **16**, and **19** achieved relatively higher 3CL^pro^ degradation efficiency. The degradation efficiency (DC_50_: <= 5 μM) of compounds **15**, **16**, and **19** was superior to that of a recently published GC-376-based PROTAC (DC_50_: 40~80 μM) [12]. Both PROTACs **15** and **16** have polyethylene glycol linkers of different lengths, which may increase the lipophilicity of the PROTACs and enhance the degradation activity. If compared with **17**, compound **18** contained a longer linker chain to obtain stronger lipophilicity, which to some extent enhanced the degradation activity. Compounds **17**, **18**, and **19** are PROTAC molecules with lenalidomide as the CRBN ligand, but compound **19** shows the best degradation activity with a DC_50_ of approximately 5 µM. It is speculated that the leucine in the small molecule dipeptide part that binds to the target protein has a smaller steric hindrance, which makes **19** more likely to bind to the substrate pocket.

## 3. Materials and Methods

### 3.1. General Information

All reagents and solvents were purchased from commercial suppliers and used without further purification. Concentration or solvent removal under reduced pressure was carried out using rotary evaporator. Analytical thin layer chromatography was performed on precoated silica gel F254 TLC plates (E. Merck KG, Darmstadt, Germany) with visualization under UV light or by iodine staining. Column chromatography was conducted on silica (Merck Silica Gel 40–63 m) or performed using a Biotage SP1 flash purification system with prepacked silica gel cartridges (Biotage, Uppsala, Sweden). NMR analyses were carried out using a JNM ECZ500R (600 MHz) manufactured by Jeol resonance. Chemical shifts are reported in parts per million. The deuterium lock signal of the sample solvent was used as a reference, and coupling constants (J) are given in hertz (Hz). The splitting pattern abbreviations are as follows: s, singlet; d, doublet; t, triplet; q, quartet; dd, doublet of doublet; td, triplet of doublet; and m, multiplet. The MS system was operated using ESI in the positive and the negative ionization mode. The optimized conditions of the QTOF-MS system for both ionization modes were as follows: drying gas temperature, 300 °C; drying gas flow, 10 L/min; nebulization pressure, 45 psi; sheath gas temperature, 350 °C; sheath gas flow, 10 L/min; capillary voltage, 3500 V; nozzle voltage, 0 V; fragmentor voltage, 175 V; and skimmer voltage, 65 V. The mass range was 50–1700 *m*/*z*, and the scan rate was 2.00 spectra/sec for both MS and MS/MS analyses.

### 3.2. Synthetic Procedures

#### 3.2.1. Synthesis of Peptidomimetic Compounds (**4a**, **4b**, **4c**, and **4d**)

Synthesis of **4a**. A mixture of N-Boc-L-cyclohexylglycine (2.57 g, 10 mmol), L-Valine methyl ester hydrochloride (1.67 g, 10 mmol) in dichloromethane (20 mL) was stirred. Under ice bath conditions, HATU (5.32 g, 14 mmol) was added and 5 mL of DIPEA was dropped in. After maintaining for 30 min, the ice bath was removed, and the mixture was stirred at room temperature for 10 h. After the reaction was completed, the product **3a** was isolated and purified by column chromatography in 81% yield. Compound **3a** (1.66 g, 4.5 mmol) was dissolved in THF (15 mL), and then lithium hydroxide monohydrate (7.55 g, 18 mmol), 30% hydrogen peroxide solution (12.24 g, 36 mmol), and water (5 mL) were added under an ice bath. The ice bath was maintained for 2 h, and then the mixture was stirred at room temperature for 6 h. After the reaction was completed, 80 mL of ethyl acetate was added for extraction. The pH was adjusted to 1 with hydrochloric acid, and the mixture was concentrated under reduced pressure to obtain **4a** in 95%. ^1^H NMR (600 MHz, DMSO) δ 12.60 (s, 1H), 7.78 (d, *J* = 7.9 Hz, 1H), 6.73 (d, *J* = 8.7 Hz, 1H), 4.14 (s, 1H), 3.87 (s, 1H), 2.03 (m, 1H), 1.61 (m, 6H), 1.37 (s, 9H), 1.02 (m, 5H), 0.87 (d, *J* = 6.8 Hz, 6H).

Synthesis of **4b**. A mixture of N-Boc-L-leucine (2.31 g, 10 mmol), L-Phenylalanine methyl ester hydrochloride (2.15 g, 10 mmol) in dichloromethane (20 mL) was stirred. Under ice bath conditions, HATU (5.32 g, 14 mmol) was added and 5 mL of DIPEA was dropped in. After maintaining for 30 min, the ice bath was removed, and the mixture was stirred at room temperature for 10 h. After the reaction was completed, the product **3b** was isolated and purified by column chromatography in 90% yield. Compound **3b** (1.77 g, 4.5 mmol) was dissolved in THF (15 mL), and then lithium hydroxide monohydrate (7.55 g, 18 mmol), 30% hydrogen peroxide solution (12.24 g, 36 mmol) and water (5 mL) were added under an ice bath. The ice bath was maintained for 2 h and then the mixture was stirred at room temperature for 6 h. After the reaction was completed, 80 mL of ethyl acetate was added for extraction. The pH was adjusted to 1 with hydrochloric acid, and the mixture was concentrated under reduced pressure to obtain **4b** in 95%.^1^H NMR (600 MHz, DMSO) δ 12.75 (s, 1H), 7.90 (d, *J* = 7.9 Hz, 1H), 7.27–7.18 (m, 5H), 6.85 (d, *J* = 8.6 Hz, 1H), 4.44 (t, *J* = 5.2 Hz, 1H), 3.98–3.90 (t, *J* = 3.6 Hz, 1H), 3.04 (d, *J* = 5.0 Hz, 1H), 2.91 (d, *J* = 8.8 Hz, 1H), 1.56–1.47 (t, *J* = 3.9 Hz, 1H), 1.39–1.25 (m, 11H), 0.82 (d, *J* = 6.6 Hz, 6H).

Synthesis of **4c**. A mixture of N-Boc-L-cyclohexylglycine (2.57 g, 10 mmol), L-Phenylalanine methyl ester hydrochloride (2.15 g, 10 mmol) in dichloromethane (20 mL) was stirred. Under ice bath conditions, HATU (5.32 g, 14 mmol) was added and 5 mL of DIPEA was dropped in. After maintaining for 30 min, the ice bath was removed, and the mixture was stirred at room temperature for 10 h. After the reaction was completed, the product **3c** was isolated and purified by column chromatography in 81% yield. Compound **3c** (1.88 g, 4.5 mmol) was dissolved in THF (15 mL), and then lithium hydroxide monohydrate (7.55 g, 18 mmol), 30% hydrogen peroxide solution (12.24 g, 36 mmol), and water (5 mL) were added under an ice bath. The ice bath was maintained for 2 h, and then the mixture was stirred at room temperature for 6 h. After the reaction was completed, 80 mL of ethyl acetate was added for extraction. The pH was adjusted to 1 with hydrochloric acid, and the mixture was concentrated under reduced pressure to obtain **4c** in 95%.^1^H NMR (600 MHz, DMSO) δ 12.72 (s, 1H), 8.02 (d, *J* = 7.7 Hz, 1H), 7.28–7.15 (m, 5H), 6.58 (d, *J* = 9.1 Hz, 1H), 4.45 (d, *J* = 4.5 Hz, 1H), 3.77 (t, *J* = 8.0 Hz, 1H), 3.05 (d, *J* = 4.1 Hz, 1H), 2.91–2.82 (d, *J* = 4.6 Hz, 1H), 1.62–1.29 (m, 15H), 1.04 (m, 3H), 0.86 (m, 2H).

Synthesis of **4d**. A mixture of N-Boc-L-phenylalanine (2.65 g, 10 mmol), L-Leucine methyl ester hydrochloride (1.81 g, 10 mmol) in dichloromethane (20 mL) was stirred. Under ice bath conditions, HATU (5.32 g, 14 mmol) and DIPEA (5 mL) were added. After maintaining for 30 min, the ice bath was removed, and the mixture was stirred at room temperature for 10 h. After the reaction was completed, the product **3d** was isolated, and purified by column chromatography in 79% yield. Compound **3d** (1.77 g, 4.5 mmol) was dissolved in THF (15 mL), and then lithium hydroxide monohydrate (7.55 g, 18 mmol), 30% hydrogen peroxide solution (12.24 g, 36 mmol), and water (5 mL) were added under an ice bath. The ice bath was maintained for 2 h, and then the mixture was stirred at room temperature for 6 h. After the reaction was completed, 80 mL of ethyl acetate was added for extraction. The pH was adjusted to 1 with hydrochloric acid, and the mixture was concentrated under reduced pressure to obtain **4d** in 95%. ^1^H NMR (600 MHz, DMSO) δ 12.58 (s, 1H), 8.11 (d, *J* = 8.0 Hz, 1H), 7.32–7.18 (m, 5H), 6.89 (d, *J* = 8.7 Hz, 1H), 4.29–4.23 (t, *J* = 3.6 Hz, 1H), 4.21–4.06 (t, *J* = 6.9 Hz, 1H), 2.96 (d, *J* = 3.7 Hz, 1H), 2.72 (d, *J* = 4.7 Hz, 1H), 1.60–1.48 (t, *J* = 6.5 Hz, 2H), 1.32–1.21 (m, 10H), 0.87 (d, *J* = 6.5 Hz, 6H).

#### 3.2.2. Synthesis of Linkers (**9a**, **9b**, **14a**, and **14b**)

Synthesis of **9a**. A mixture of 4-flurothalidomide (**7**, 0.83 g, 3 mmol), Diethylene glycol amine (0.63 g, 6 mmol), and DIPEA (1 mL) in DMF (10 mL) was stirred and heated to reflux at 145 °C for 4 h. After the reaction was completed, the solvent was removed under reduced pressure, and the residue was extracted with 50 mL of ethyl acetate. The extract was washed twice each with saturated NaHCO_3_ solution and saturated NaCl solution, and the product **9a** was obtained after column chromatography separation, resulting in **9a** in 58% yield. ^1^H NMR (600 MHz, DMSO) δ 11.11 (s, 1H), 7.59 (s, 1H), 7.14 (s, 1H), 7.05 (s, 1H), 6.62 (s, 1H), 5.12–4.98 (m, 1H), 4.63 (t, *J* = 5.4 Hz, 1H), 3.73–3.26 (m, 12H), 2.66–2.41 (m, 4H), 1.99 (s, 2H).

Synthesis of **9b**. A mixture of 4-flurothalidomide (**7**, 0.83 g, 3 mmol), Triethylene glycol amine (0.89 g, 6 mmol), and DIPEA (1 mL) in DMF (10 mL) was stirred and heated to reflux at 145 °C for 4 h. After the reaction was completed, the solvent was removed under reduced pressure, and the residue was extracted with 50 mL of ethyl acetate. The extract was washed twice each with saturated NaHCO_3_ solution and saturated NaCl solution, and the product 9b was obtained after column chromatography separation, resulting in **9b** in 68% yield. ^1^HNMR (600 MHz, DMSO) δ 11.10 (s, 1H), 7.58 (t, *J* = 7.7 Hz, 1H), 7.15 (d, *J* = 8.6 Hz, 1H), 7.04 (d, *J* = 7.0 Hz, 1H), 6.61 (s, 1H), 5.05 (dd, *J* = 12.7, 5.1 Hz, 1H), 4.56 (t, *J* = 5.1 Hz, 1H), 3.61 (d, *J* = 5.1 Hz, 2H), 3.58–3.51 (m, 4H), 3.47 (d, *J* = 4.9 Hz, 4H), 3.41 (t, *J* = 5.0 Hz, 2H), 2.61–2.52 (m, 4H).

Synthesis of **14a**. A mixture of lenalidomide (5.18 g, 20 mmol), Succinic anhydride (2.40 g, 24 mmol) in Acetonitrile (60 mL), and DMF (20 mL) was stirred and heated to reflux at 80 °C for 4 h. After natural cooling to room temperature, the product **12** was obtained by suction filtration under normal pressure in 69% yield.

A mixture of **12** (1.08 g, 3 mmol), Monoboc butylenediamine (0.56 g, 3 mmol), and DMAP (0.18, 1.5 mmol) in DMF (15 mL) was stirred. Under ice bath conditions, EDCI (0.86 g, 4.5 mmol) and DIPEA (1.5 mL) were added. After maintaining for 30 min, the ice bath was removed, and the mixture was stirred at room temperature for 10 h. After the reaction was completed, the product **14a** was isolated and purified by column chromatography, resulting in **14a** 57% yield. ^1^HNMR (600 MHz, DMSO) δ 11.04 (s, 1H), 9.87 (s, 1H), 7.85 (d, *J* = 33.3 Hz, 2H), 7.49 (s, 2H), 6.79 (s, 1H), 5.15 (dd, *J* = 13.3, 5.1 Hz, 1H), 4.35 (dd, *J* = 42.8, 17.5Hz, 2H), 3.01 (d, *J* = 5.2 Hz, 2H), 2.88 (d, *J* = 5.1 Hz, 3H), 2.60 (dd, *J* = 17.4, 10.5 Hz, 3H),2.40 (t, *J* = 7.3 Hz, 2H), 2.32 (ddd, *J* = 26.3, 13.3, 4.3 Hz, 1H), 2.03 (dd, *J* = 8.9, 3.6 Hz, 1H), 1.35 (d, *J* = 12.4 Hz, 13H).

Synthesis of **14b**. A mixture of **12** (1.08 g, 3 mmol), Monoboc hexamediamine (0.65 g, 3 mmol), and DMAP (0.18, 1.5 mmol) in DMF (15 mL) was stirred. Under ice bath conditions, EDCI (0.86 g, 4.5 mmol) and DIPEA (1.5 mL) were added. After maintaining for 30 min, the ice bath was removed, and the mixture was stirred at room temperature for 10 h. After the reaction was completed, the product **14b** was isolated and purified by column chromatography, resulting in **14b** 64% yield. ^1^HNMR (600 MHz, DMSO) δ 11.03 (s, 1H), 9.85 (s, 1H), 7.83 (dd, *J* = 12.3, 5.2 Hz, 2H), 7.49 (s, 2H), 6.76 (s, 1H), 5.15 (dd, *J* = 13.3, 5.1 Hz, 1H), 4.35 (dd, *J* = 40.9, 17.5 Hz, 2H), 3.01 (dd, *J* = 12.9, 6.7 Hz, 2H), 2.87 (dd, *J* = 13.2, 6.7 Hz, 2H), 2.59 (t, *J* = 7.1 Hz, 2H), 2.41 (t, *J* = 7.3 Hz, 2H), 2.32 (qd, *J* = 13.3, 4.5 Hz, 1H), 2.07–2.00 (m, 1H), 1.38–1.20 (m, 19H).

#### 3.2.3. Synthesis of PROTACs (**15**–**19**)

Synthesis of **15**. A mixture of **9a** (0.41 g, 1 mmol) and **4a** (0.39 g, 1 mmol) in dichloromethane (20 mL) was stirred. Under ice bath conditions, HOAT (5.32 g, 14 mmol), EDCI (0.29 g, 1.5 mmol), and DIPEA (0.5 mL) were added. After maintaining for 30 min, the ice bath was removed, and the mixture was stirred at room temperature for 10 h. After the reaction was completed, the solvent was removed under reduced pressure, and the residue was extracted with 50 mL of ethyl acetate. The extract was washed twice each with saturated NaHCO_3_ solution and saturated NaCl solution, and the product **9b** was obtained after column chromatography separation, resulting in **15** in 37% yield. ^1^H NMR (600 MHz, DMSO) δ 11.11 (s, 1H), 8.22 (s, 1H), 7.78 (d, *J* = 8.4 Hz, 1H), 7.59 (t, *J* = 7.8 Hz, 1H), 7.15 (d, *J* = 5.0 Hz, 1H), 7.05 (d, *J* = 7.0 Hz, 1H), 6.62 (s, 1H), 5.06 (d, *J* = 5.4 Hz, 1H), 4.27–4.17 (m, 2H), 4.15 (d, *J* = 5.8 Hz, 1H), 4.03 (q, *J* = 7.1 Hz, 1H), 3.70–3.60 (m, 4H), 3.52–3.43 (t, *J* = 5.8 Hz, 2H), 2.58 (m, 2H), 2.03 (m, 2H), 1.65 (m, 2H), 1.37 (d, *J* = 6.4 Hz, 9H), 1.32–1.05 (m, 10H), 0.86 (d, *J* = 5.5 Hz, 6H).^13^C NMR (151 MHz, DMSO) δ 172.80 (s), 171.45 (s), 170.09 (s), 168.91 (s), 167.27 (s), 162.10 (s), 155.32 (s), 146.37 (s), 136.21 (s), 132.08 (s), 117.46 (s), 110.70 (s), 109.27 (s), 77.95 (s), 68.79 (s), 68.05 (s), 62.57 (s), 59.75 (s), 59.06 (s), 56.97 (s), 48.54 (s), 41.58 (s), 30.98 (s), 29.95 (s), 29.81 (s), 29.08 (s), 28.17 (s), 25.83 (s), 25.55 (s), 22.13 (s), 20.76 (s), 19.03 (s), 17.97 (s). HRESIMS: *m*/*z* calcd. for C_35_H_49_N_5_O_10_ [M + Na] + 722.3377, found 722.3378.

Synthesis of **16**. A mixture of **9b** (0.45 g, 1 mmol) ab=nd **4b** (0.42 g, 1 mmol) in dichloromethane (20 mL) was stirred. Under ice bath conditions, HOAT (5.32 g, 14 mmol), EDCI (0.29 g,1.5 mmol), and DIPEA (0.5 mL) were added. After maintaining for 30 min, the ice bath was removed and the mixture was stirred at room temperature for 10 h. After the reaction was completed, the solvent was removed under reduced pressure, and the residue was extracted with 50 mL of ethyl acetate. The extract was washed twice each with saturated NaHCO_3_ solution and saturated NaCl solution, and the product **9b** was obtained after column chromatography separation, resulting in **16** in 31% yield. ^1^H NMR (600 MHz, DMSO) δ 11.10 (s, 1H), 8.67 (d, *J* = 6.2 Hz, 0.5H), 8.66 (d, *J* = 6.2 Hz, 0.5H), 8.18 (d, *J* = 7.7 Hz, 0.5H), 8.17 (d, *J* = 7.7 Hz, 0.5H), 7.59–7.55 (t, *J* = 4.9 Hz, 1H), 7.52 (d, *J* = 4.4 Hz, 1H), 7.26–7.17 (m, 5H), 7.12 (d, *J* = 8.6 Hz, 1H), 7.04 (d, *J* = 7.0 Hz, 1H), 5.06 (d, *J* = 5.4 Hz, 1H), 4.55–4.43 (t, *J* = 4.9 Hz, 1H), 4.21–4.06 (t, *J* = 4.4 Hz, 2H), 3.97 (t, *J* = 8.9 Hz, 1H), 3.64–3.44 (m, 10H), 3.07–2.88 (t, *J* = 4.4 Hz, 2H), 2.04–1.99 (m, 1H), 1.58–1.41 (m, 1H), 1.36–1.10 (m, 14H), 0.85–0.76 (m, 6H). ^13^C NMR (151 MHz, DMSO) δ 172.82 (s), 172.60 (s), 171.44 (s), 170.09 (s), 168.93 (s), 167.29 (s), 151.08 (s), 146.38 (s), 137.03 (s), 136.21 (s), 132.08 (s), 129.20 (s), 128.85 (s), 128.14 (s), 126.49 (s), 120.71 (s), 117.41 (s), 110.67 (s), 109.23 (s), 77.89 (s), 69.72 (s), 68.92 (s), 68.17 (s), 63.90 (s), 53.28 (s), 53.28 (s), 52.40 (s), 48.55 (s), 41.68 (s), 36.62 (s), 31.00 (s), 28.18 (s), 24.10 (s), 22.88 (s), 22.88 (s), 22.15 (s), 21.55 (s). HRESIMS: *m*/*z* calcd. for C_39_H_51_N_5_O_11_ [M + Na] + 788.3483, found 788.3486.

Synthesis of **17**. A mixture of **14a** (0.51 g, 1 mmol) in Trifluoroacetic acid (8 mL) was stirred. After stirring at room temperature for 2 h, the solvent was removed under reduced pressure. Compound **4c** (0.44 g, 1 mmol) was added and dissolved in DMF (8 mL), and then diphenylphosphinoyl chloride (0.24 g, 1 mmol) and DIPEA (0.5 mL) were added dropwise in sequence in an ice bath. After maintaining for 30 min, the ice bath was removed, and the mixture was stirred at room temperature for 10 h. After the reaction was completed, the solvent was removed under reduced pressure, and the residue was extracted with 50 mL of ethyl acetate. The extract was washed twice each with saturated NaHCO_3_ solution and saturated NaCl solution, and the product **17** was obtained after column chromatography separation, resulting in **17** in 41% yield. ^1^H NMR (600 MHz, DMSO) δ 11.04 (s, 1H), 9.92 (s, 1H), 9.24 (s, 1H), 8.34 (d, *J* = 8.5 Hz, 1H), 7.95–7.81 (m, 3H), 7.48 (q, *J* = 7.4 Hz, 2H), 7.24–7.12 (m, 5H), 5.15 (d, *J* = 4.9 Hz, 1H), 4.40 (d, *J* = 7.5 Hz, 1H), 4.32 (d, *J* = 7.5 Hz, 1H), 3.69 (t, *J* = 7.6 Hz, 1H), 3.59 (d, *J* = 7.0 Hz, 2H), 3.18–2.75 (m, 8H), 2.65–2.54 (m, 3H), 2.42 (s, 2H), 2.32 (m, 1H), 2.09–1.96 (m, 1H), 1.43–1.21 (m, 23H). ^13^C NMR (151 MHz, DMSO) δ 172.86 (s), 171.53 (s), 171.05 (s), 170.96 (s), 170.73 (s), 170.65 (s), 167.84 (s), 155.38 (s), 137.65 (s), 133.84 (s), 133.47 (s), 132.62 (s), 129.15 (s), 128.60 (s), 127.94 (s), 126.16 (s), 124.96 (s), 118.84 (s), 78.17 (s), 78.13 (s), 59.76 (s), 53.69 (s), 51.51 (s), 46.46 (s), 41.59 (s), 38.17 (s), 31.34 (s), 31.20 (s), 30.43 (s), 28.88 (s), 28.15 (s), 26.56 (s), 26.41 (s), 25.71 (s), 25.56 (s), 22.70 (s), 17.98 (s), 16.70 (s), 12.24 (s). HRESIMS: *m*/*z* calcd. for C_43_H_57_N_7_O_9_ [M + Na] + 838.4115, found 838.4118.

Synthesis of **18**. A mixture of **14b** (0.54 g, 1 mmol) in Trifluoroacetic acid (8 mL) was stirred. After stirring at room temperature for 2 h, the solvent was removed under reduced pressure. Compound **4c** (0.44 g, 1 mmol) was added and dissolved in DMF (8 mL), and then diphenylphosphinoyl chloride (0.24 g,1 mmol) and DIPEA (0.5 mL) were added dropwise in sequence in an ice bath. After maintaining for 30 min, the ice bath was removed, and the mixture was stirred at room temperature for 10 h. After the reaction was completed, the solvent was removed under reduced pressure, and the residue was extracted with 50 mL of ethyl acetate. The extract was washed twice each with saturated NaHCO_3_ solution and saturated NaCl solution, and the product **18** was obtained after column chromatography separation, resulting in 18 in 44% yield. ^1^H NMR (600 MHz, DMSO) δ 11.04 (s, 1H), 9.93 (s, 1H), 7.88 (s, 3H), 7.83 (d, *J* = 7.1 Hz, 1H), 7.49 (t, *J* = 8.0 Hz, 2H), 7.25–7.18 (m, 4H), 7.15 (t, *J* = 6.8 Hz, 1H), 6.75 (d, *J* = 8.7 Hz, 1H), 5.15 (d, *J* = 5.1 Hz, 1H), 4.36 (q, *J* = 8.7 Hz, 2H), 3.73–3.55 (m, 2H), 3.11 (d, *J* = 4.0 Hz, 1H), 3.01 (d, *J* = 6.0 Hz, 3H), 2.94 (d, *J* = 8.7 Hz, 3H), 2.79 (d, *J* = 9.3 Hz, 1H), 2.65–2.56 (m, 3H), 2.42 (t, *J* = 7.2 Hz, 2H), 2.32 (d, *J* = 4.2 Hz, 1H), 2.07–1.98 (m, 1H), 1.51 (m, 5H), 1.37 (s, 10H), 1.32–1.25 (m, 12H). ^13^C NMR (151 MHz, DMSO) δ 172.84 (s), 171.04 (s), 170.89 (s), 170.72 (s), 170.54 (s), 167.84 (s), 155.38 (s), 137.64 (s), 133.84 (s), 133.48 (s), 132.61 (s), 129.14 (s), 128.58 (s), 127.92 (s), 126.16 (s), 124.95 (s), 118.83 (s), 78.11 (s), 59.75 (s), 54.92 (s), 53.70 (s), 53.29 (s), 51.49 (s), 46.46 (s), 41.57 (s), 38.47 (s), 37.89 (s), 31.20 (s), 30.46 (s), 28.87 (s), 28.16 (s), 26.05 (s), 26.05 (s), 25.70 (s), 22.70 (s), 17.96 (s), 16.69 (s), 12.21 (s). HRESIMS: *m*/*z* calcd. for C_45_H_61_N_7_O_9_ [M + Na] + 866.4428, found 866.4434.

Synthesis of **19**. A mixture of **14b** (0.54 g, 1 mmol) in Trifluoroacetic acid (8 mL) was stirred. After stirring at room temperature for 2 h, the solvent was removed under reduced pressure. Compound **4d** (0.42 g, 1 mmol) was added and dissolved in DMF (8 mL), and then diphenylphosphinoyl chloride (0.24 g,1 mmol) and DIPEA (0.5 mL) were added dropwise in sequence in an ice bath. After maintaining for 30 min, the ice bath was removed, and the mixture was stirred at room temperature for 10 h. After the reaction was completed, the solvent was removed under reduced pressure, and the residue was extracted with 50 mL of ethyl acetate. The extract was washed twice each with saturated NaHCO_3_ solution and saturated NaCl solution, and the product **19** was obtained after column chromatography separation, resulting in **19** in 80% yield. ^1^H NMR (600 MHz, DMSO) δ 11.04 (s, 1H), 9.90 (s, 1H), 8.03–7.77 (m, 4H), 7.49 (t, *J* = 8.1 Hz, 2H), 7.24 (d, *J* = 3.3 Hz, 4H), 7.18 (d, *J* = 4.4 Hz, 1H), 6.96 (d, *J* = 8.4 Hz, 1H), 5.15 (d, *J* = 5.0 Hz, 1H), 4.36 (q, *J* = 8.7 Hz, 2H), 4.27 (d, *J* = 8.6 Hz, 1H), 4.16 (d, *J* = 4.7 Hz, 1H), 3.63–3.55 (m, 1H), 3.11 (d, *J* = 4.1 Hz, 1H), 2.98 (m, 6H), 2.73 (d, *J* = 9.4 Hz, 1H), 2.59 (d, *J* = 6.7 Hz, 3H), 2.41 (t, *J* = 7.2 Hz, 2H), 2.32 (d, *J* = 4.2 Hz, 1H), 2.06–1.99 (m, 1H), 1.38–1.25 (m, 18H), 0.85 (d, *J* = 6.4 Hz, 6H). ^13^C NMR (151 MHz, DMSO) δ 172.84 (s), 171.53 (s), 171.24 (s), 171.04 (s), 170.89 (s), 170.71 (s), 167.83 (s), 155.23 (s), 138.09 (s), 133.83 (s), 133.47 (s), 132.61 (s), 129.18 (s), 128.59 (s), 127.97 (s), 126.14 (s), 124.95 (s), 118.84 (s), 78.09 (s), 55.77 (s), 53.33 (s), 51.25 (s), 51.25 (s), 46.43 (s), 41.60 (s), 41.41 (s), 38.45 (s), 37.11 (s), 31.20 (s), 30.44 (s), 29.13 (s), 28.07 (s), 26.08 (s), 24.04 (s), 22.99 (s), 22.69 (s), 21.75 (s), 17.98 (s), 16.69 (s), 12.25 (s). HRESIMS: *m*/*z* calcd. for C_43_H_59_N_7_O_9_ [M + Na] + 840.4272, found 840.4280.

### 3.3. Cell Culture and Materials

HEK293 cells were maintained at 37 °C in a humidified atmosphere with 5% CO_2_. The cells were grown in Dulbecco’s Modified Eagle Medium (DMEM, Gibco, Grand Island, NY, USA), which was supplemented with 10% fetal bovine serum (Gibco), penicillin-streptomycin (Invitrogen, Waltham, MA, USA), and non-essential amino acids (Invitrogen).

### 3.4. Construction of HEK293 Cell with Stable SARS-CoV-2 3CL^pro^ Expression

To obtain HEK293 cells stably expressing 3CL^pro^ for degrader screening, HEK293 cells were seeded at 70% confluence into 24-well plates pre-coated for 60 min with a 0.1 mg/mL solution of gelatin. The next day, the cells were transfected with 500 ng of plasmid of pcDNA3.1-3CL-c-flag using Lipofectamine 8000 (Beyotime, Nantong, China) according to the manufacturer’s instructions. After 48 h, the transfected cells were selected with 0.8 mg/mL of G418 (Beyotime, Nantong, China) for one week. Then, the surviving cells were diluted to 10 cells per ml and inoculated with 100 µL of the cell suspension per well into a 96-well plate. After approximately 10–14 days, single clones were formed and passaged to a 24-well plate, then further expanded in a 6-well plate. The cell lystes derived from single colonies were prepared with RIPA lysis buffer, and the 3CL^pro^ expressing clones (positive) were identified using Western blot.

Briefly, the protein samples were analyzed by 10% SDS-PAGE gel electrophoresis and then electro-transferred onto a PVDF membrane. After blocking with 5% skim milk for 60 min, the membrane was incubated with mouse anti-FLAG-HRP antibody (Beyotime, Nantong, China) at 1:3000 at room temperature for 1 h, then washed with TBST for 10 min three times. Protein signals were exposed using ECL luminescent solution and visualized using the fusion FX spectra System (VILBER). A signal band of approximately 27 kd indicates the 3CL^pro^ protein expression of a single clone, which suggests successful construction of a stable 3CL^pro^ expressing clone.

### 3.5. Evaluation of the 3CL^pro^ Degradation Effects of PROTAC Compounds in 3CL^pro^ Stable Expression Cell Lines

The compounds were initially formulated as 100 mM stock solutions in DMSO and were diluted to the designated concentrations using the culture medium and applied to the cell culture plates seeded the previous day. DMSO served as negative control. After 72 h, the cells were harvested and lysed with RIPA for Western blot analysis, as described above. For detection of the internal reference β-actin, anti-β-actin-HRP monoclonal antibody (Sigma, St. Louis, MO, USA) was used at a 1:10000 dilution. After the exposure of the PVDF membrane, signal bands of the β-actin and 3CL^pro^-flag were obtained. The optical density values (IOD) of the bands were analyzed using Gelpro32 software and then compared with the control. If the 3CL^pro^-flag/β-actin ratio decreased compared to the control group, it suggested a potential degradation effect of a compound on 3CL^pro^.

### 3.6. Cytotoxicity Assay

HEK293 cells seeded in 96-well plates were incubated with a series of concentrations of compounds in triplicates for 3 days. Cell viability was assessed using the Cell Counting Kit-8 (dojindo), following the manufacturer’s instructions. The absorbance was recorded at 450 nm to determine the CC_50_. CC_50_ values were calculated using a nonlinear regression analysis of data of cell viability using GraphPad Prism software 5.01.

## 4. Conclusions

We have successfully synthesized five 3CL^pro^ PROTACs with prominent intracellular degradation activity. Of the five PROTACs, **15**, **16**, and **19** exhibited excellent activities, showing DC_50_ values lower than 10 µM, while none of the compounds showed obvious cytotoxicity during the DC_50_ assay process. All PROTACs except **16** showed very low cytotoxicity, which grants the degraders potential application prospects.

## Data Availability

The original contributions presented in this study are included in the article. Further inquiries can be directed to the corresponding author(s).

## Supplementary Materials

The following supporting information can be downloaded at https://www.mdpi.com/article/10.3390/ijms26083903/s1.
