# Supplementary material for: Development of Peptidomimetic PROTACs as Potential Degraders of 3-Chymotrypsin-like Protease of SARS-CoV-2
Source: Int J Mol Sci. 2025 Apr 21;26(8):3903. doi: 10.3390/ijms26083903 (PMC12027948; doi:10.3390/ijms26083903)
Supplement: Supplementary file 1 [file ijms-26-03903-s001.zip › ijms-3561200-supplementary.pdf]

Supporting information

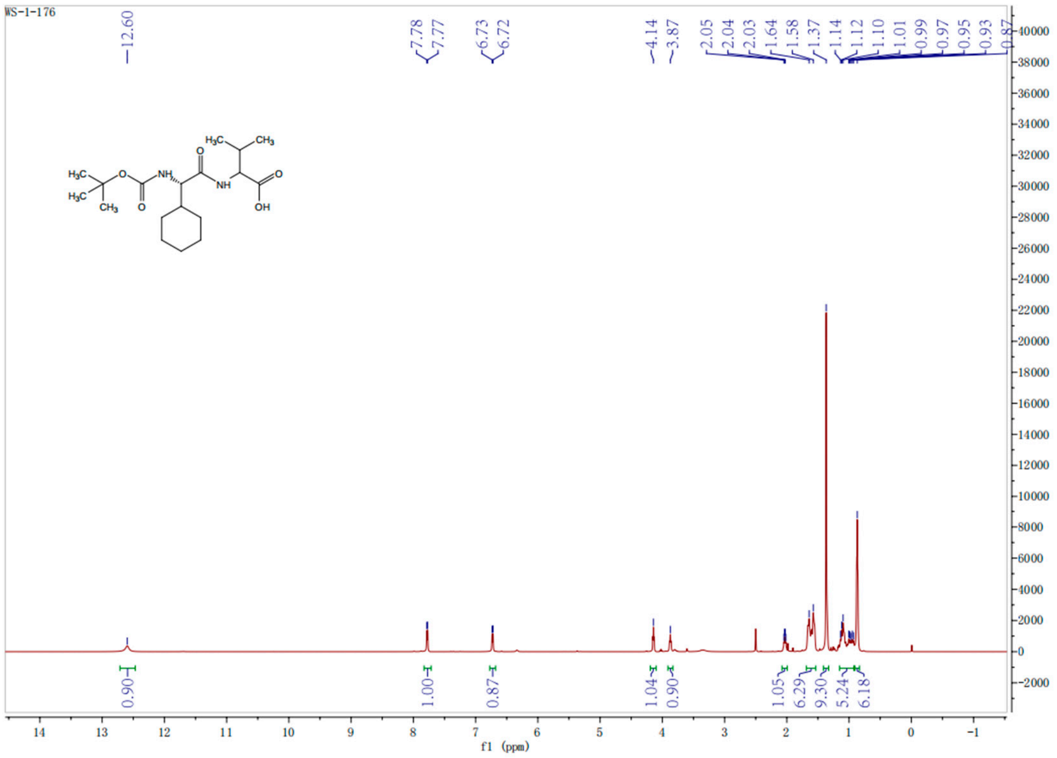

Figure S1: ^{1}H spectrum of compound **4a** (600 MHz, DMSO)

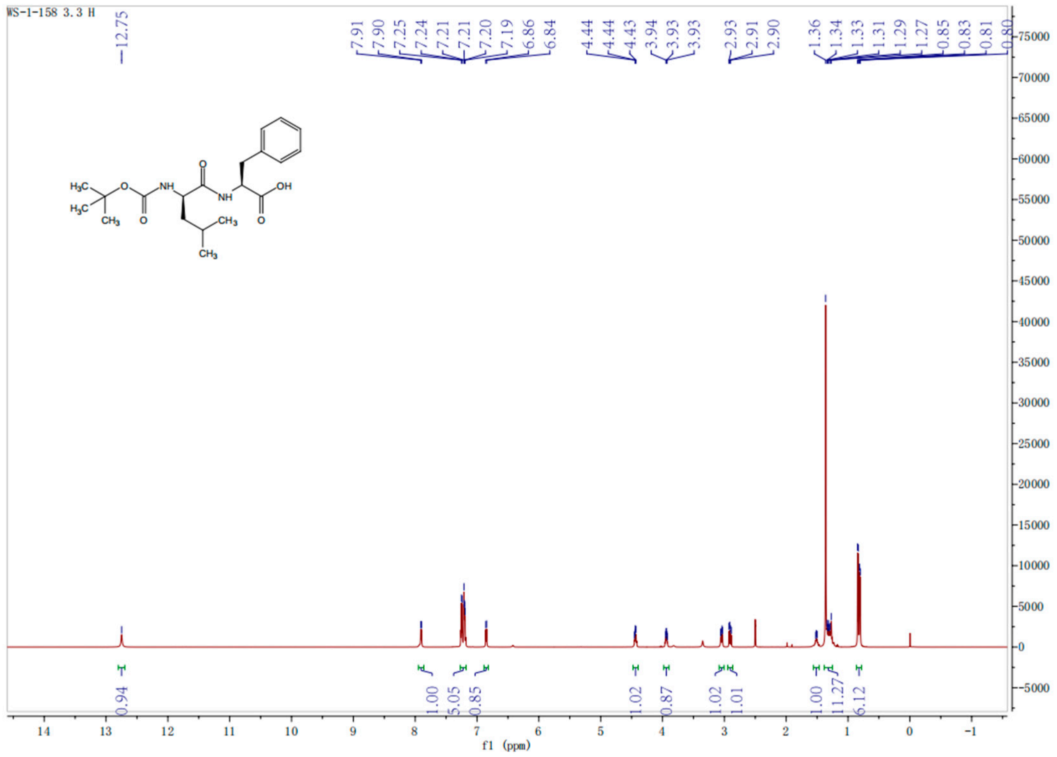

Figure S2: ^{1}H spectrum of compound **4b** (600 MHz, DMSO)

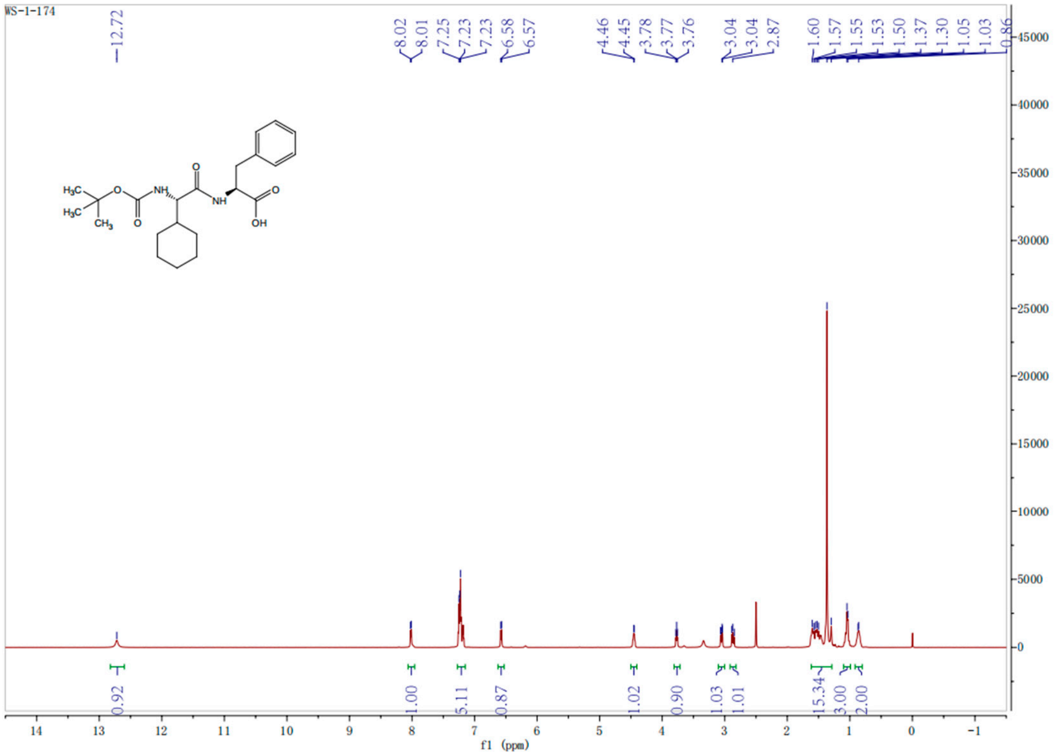

Figure S3: ^{1}H spectrum of compound **4c** (600 MHz, DMSO)

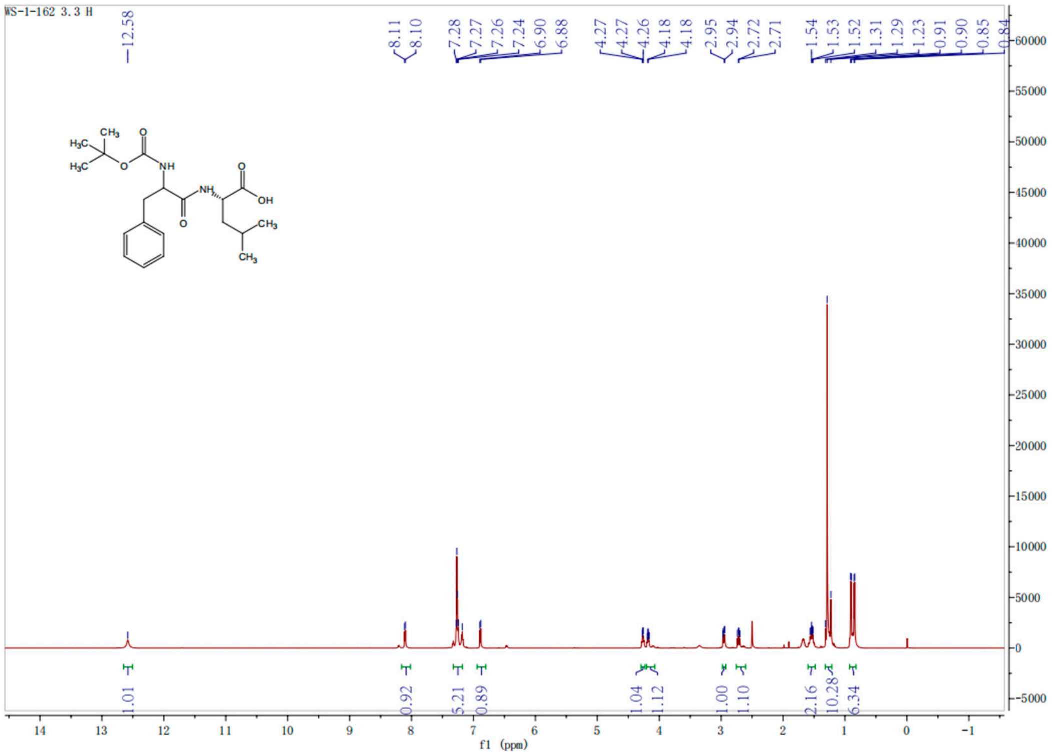

Figure S4:^{1}H spectrum of compound **4d** (600 MHz, DMSO)

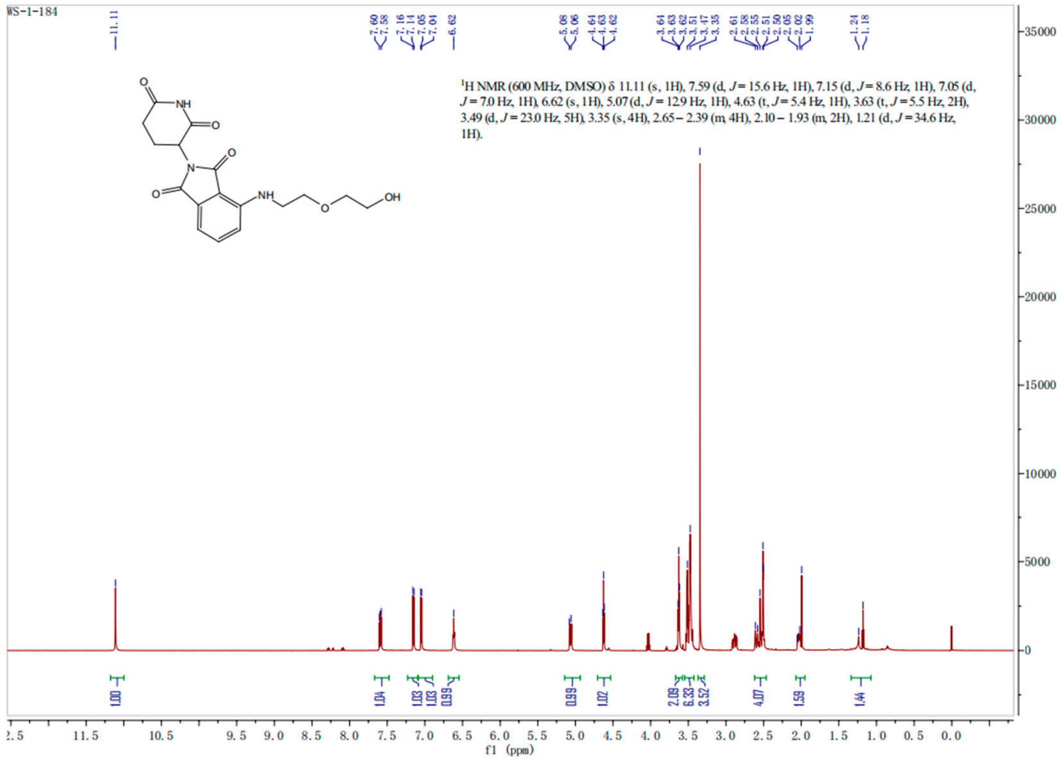

Figure S5: ^{1}H spectrum of compound **9a** (600 MHz, DMSO)

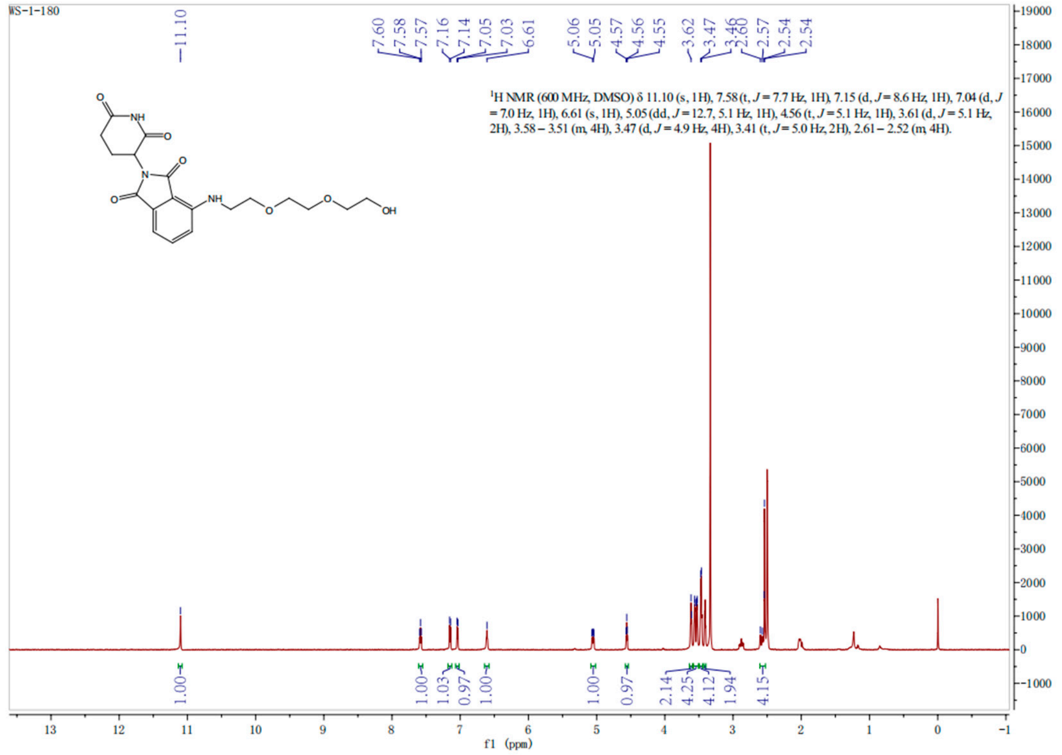

Figure S6: ^{1}H spectrum of compound **9b** (600 MHz, DMSO)

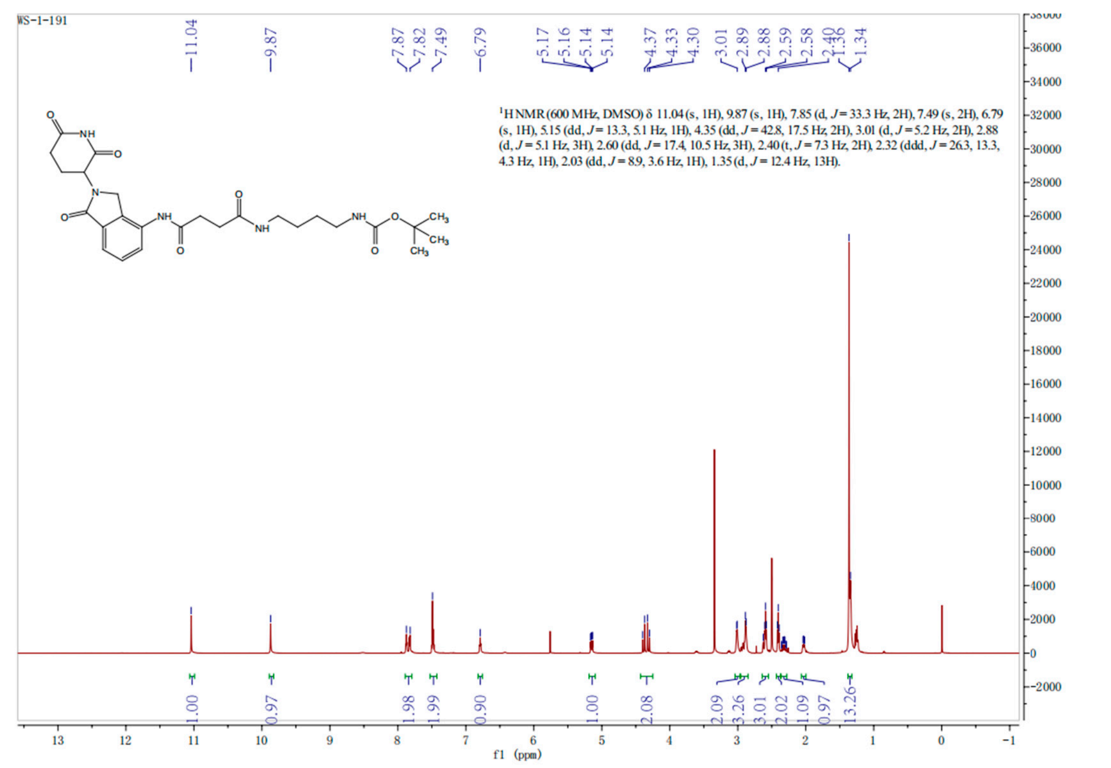

Figure S7: ^{1}H spectrum of compound **14a** (600 MHz, DMSO)

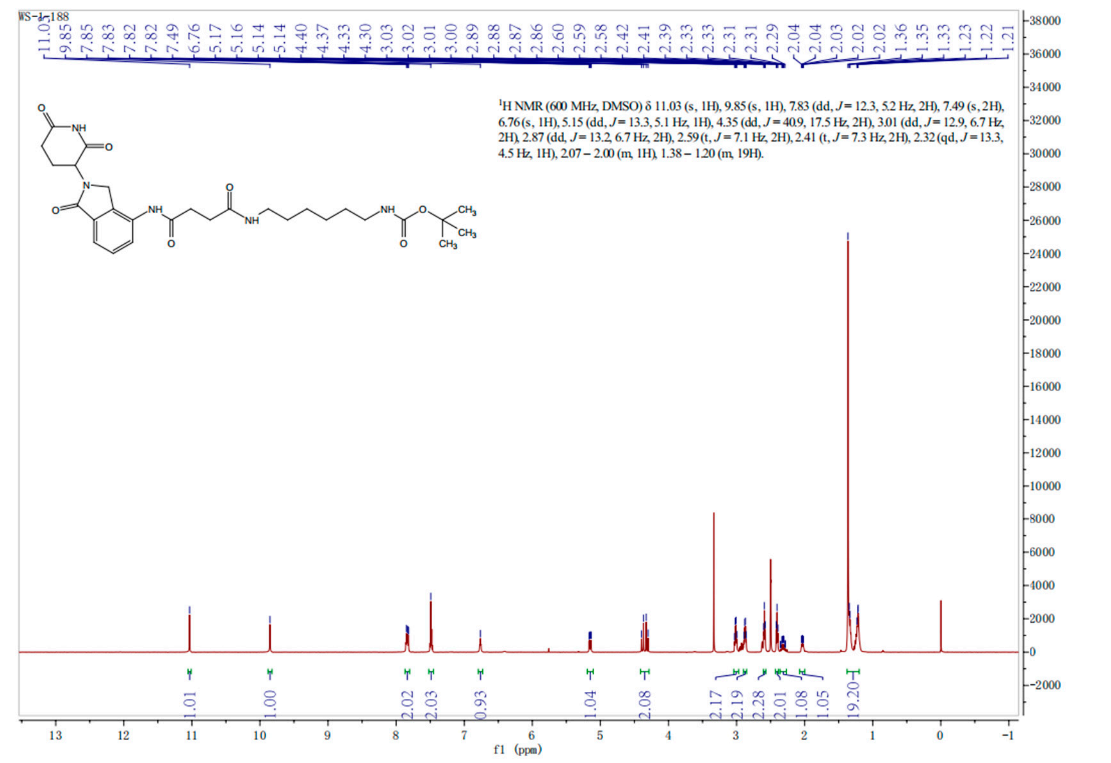

Figure S8: ^{1}H spectrum of compound **14b** (600 MHz, DMSO)

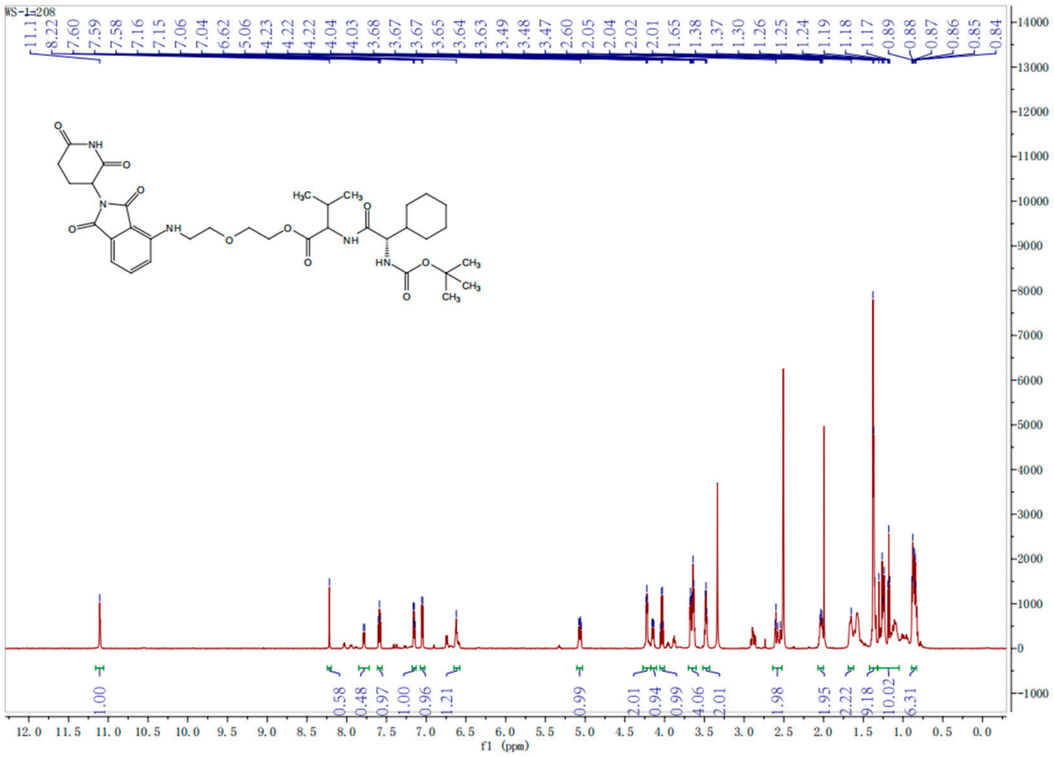

Figure S9: ^{1}H spectrum of compound **15** (600 MHz, DMSO)

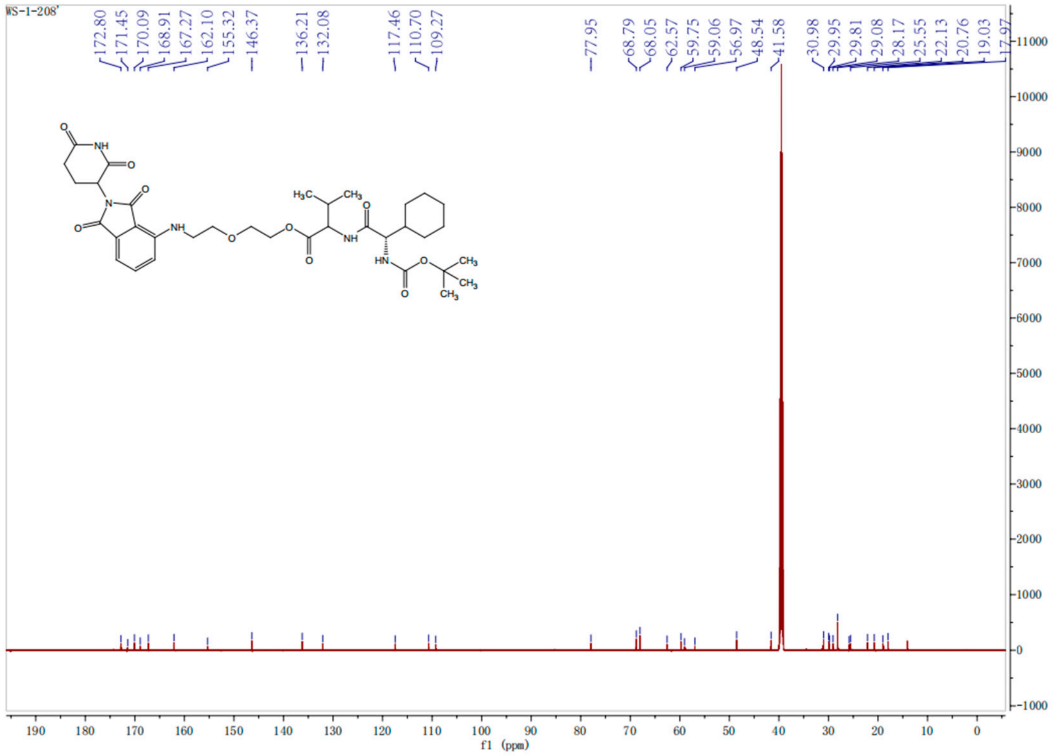

Figure S10: ^{13}C spectrum of compound **15** (600 MHz, DMSO)

Elemental Composition Report **Page 1**

Single Mass Analysis
Tolerance = 20.0 PPM / DBE: min = -1.5, max = 50.0
Element prediction: Off
Number of isotope peaks used for i-FIT = 3

Monoisotopic Mass, Even Electron Ions
1242 formula(e) evaluated with 1 results within limits (up to 50 closest results for each mass)
Elements Used:
C: 35-35 H: 49-49 N: 0-20 O: 0-20 Na: 0-3
5
230411-1-332-1-W8-1-208 6 (0.085) 1: TOF MS ES+ 2.39e+006

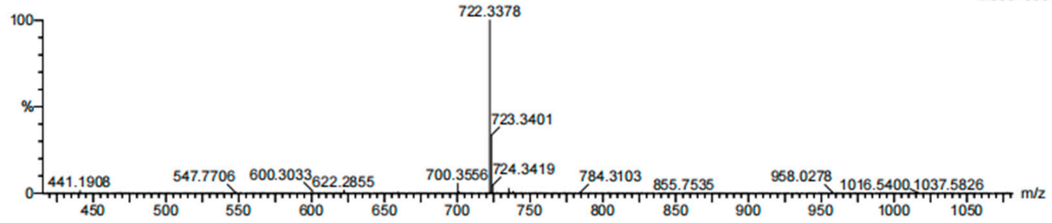

Minimum:				-1.5				
Maximum:		5.0	20.0	50.0				
Mass	Calc. Mass	mDa	PPM	DBE	i-FIT	Norm	Conf(%)	Formula
722.3378	722.3377	0.1	0.1	13.5	132.1	n/a	n/a	C35 H49 N5 O10 Na

Figure S11: Mass spectrum of compound **15**

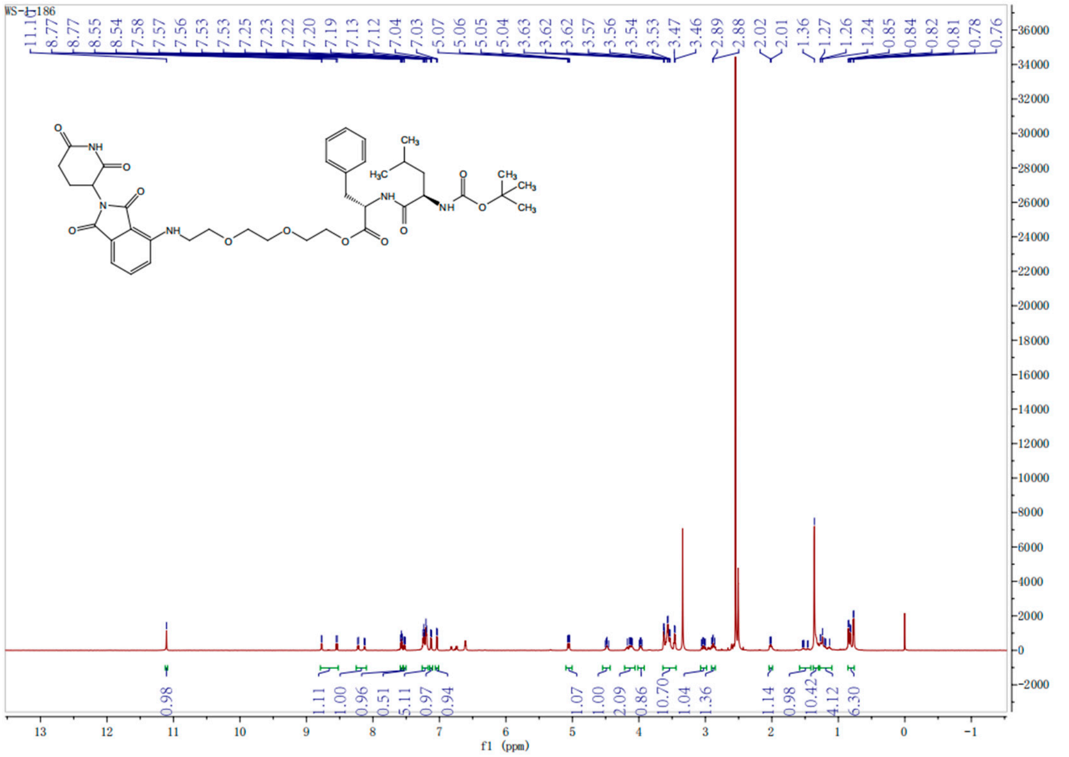

Figure S12: ^{1}H spectrum of compound **16** (600 MHz, DMSO)

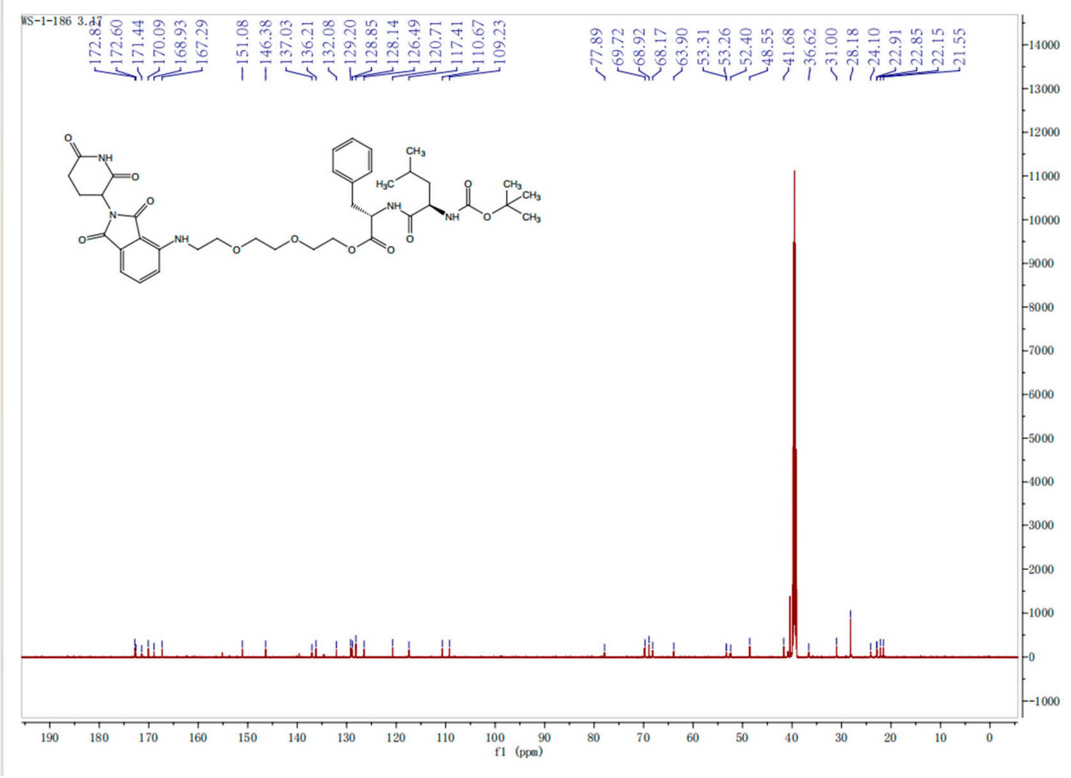

Figure S13:^{13}C spectrum of compound **16** (600 MHz, DMSO)

Elemental Composition Report **Page 1**

Single Mass Analysis

Tolerance = 20.0 PPM / DBE: min = -1.5, max = 50.0

Element prediction: Off

Number of isotope peaks used for i-FIT = 3

Monoisotopic Mass, Even Electron Ions

1173 formula(e) evaluated with 1 results within limits (up to 50 closest results for each mass)

Elements Used:

C: 39-39 H: 51-51 N: 0-20 O: 0-20 Na: 0-3

5

230411-1-332-1-W8-1-186 4 (0.059) 1: TOF MS ES+ 2.76e+006

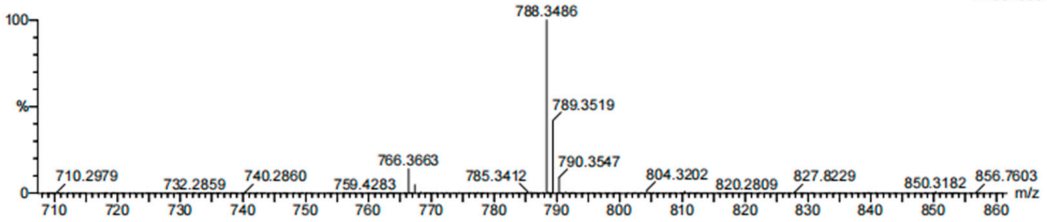

Minimum:				-1.5				
Maximum:		5.0	20.0	50.0				
Mass	Calc. Mass	mDa	PPM	DBE	i-FIT	Norm	Conf(%)	Formula
788.3486	788.3483	0.3	0.4	16.5	147.0	n/a	n/a	C39 H51 N5 O11 Na

Figure S14: Mass spectrum of compound **16**

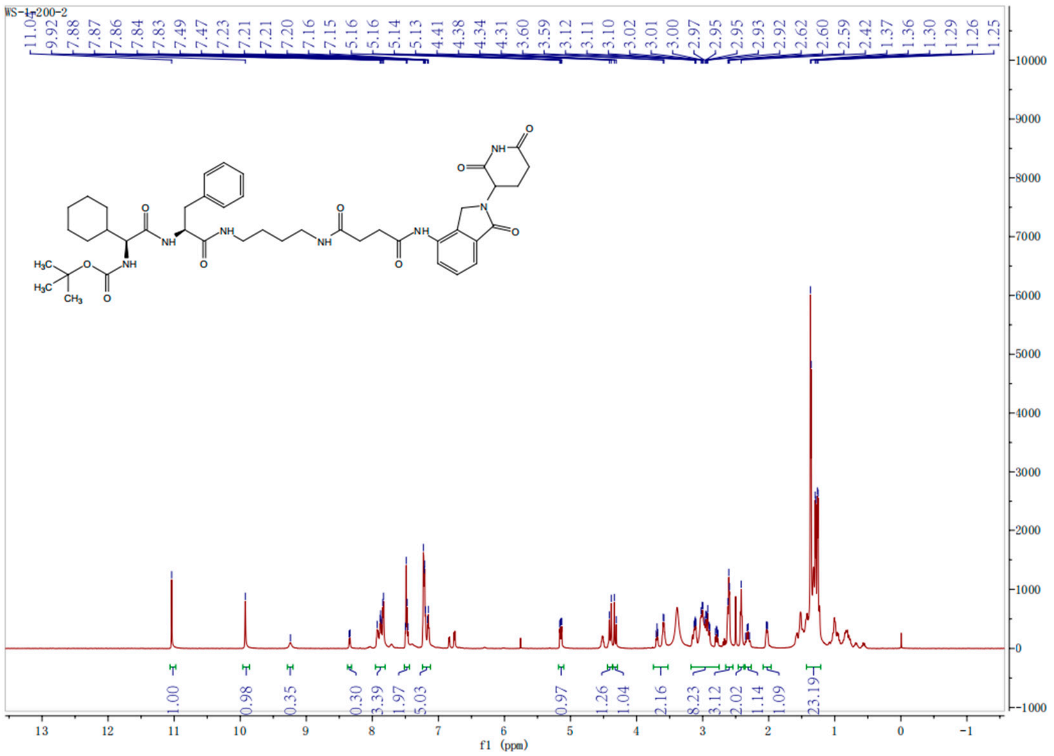

Figure S15: ^{1}H spectrum of compound **17** (600 MHz, DMSO)

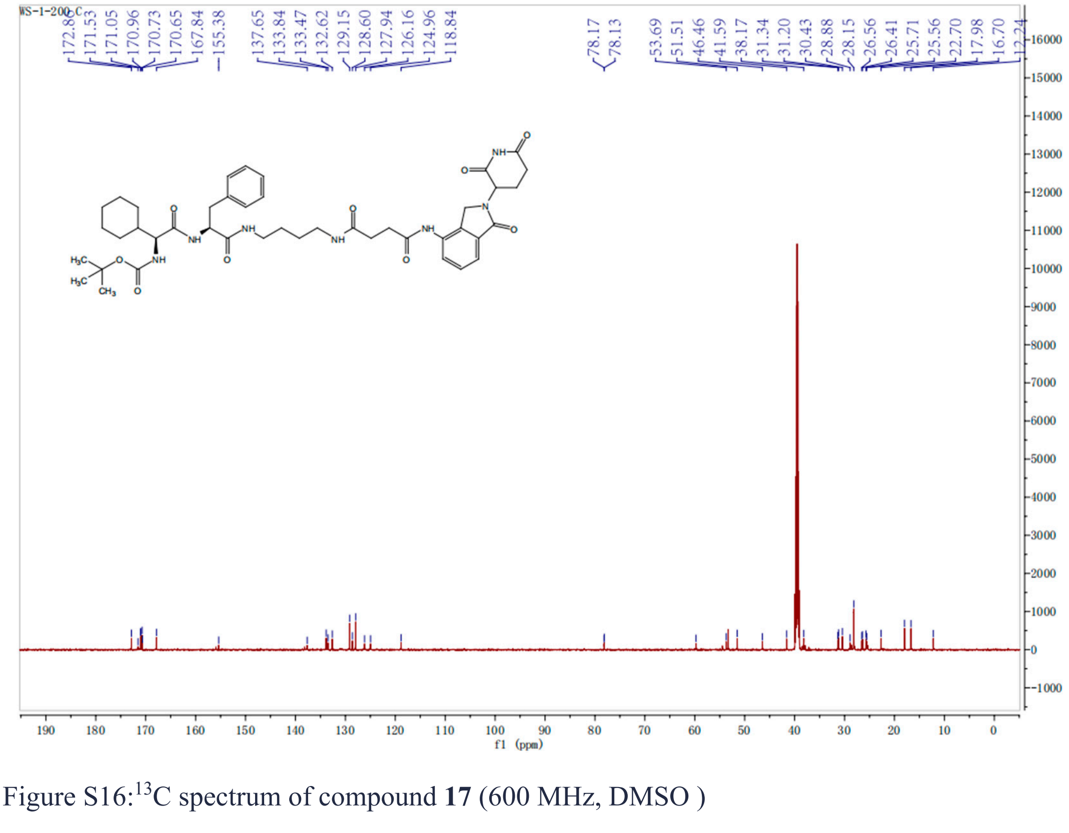

Figure S16:^{13}C spectrum of compound **17** (600 MHz, DMSO)

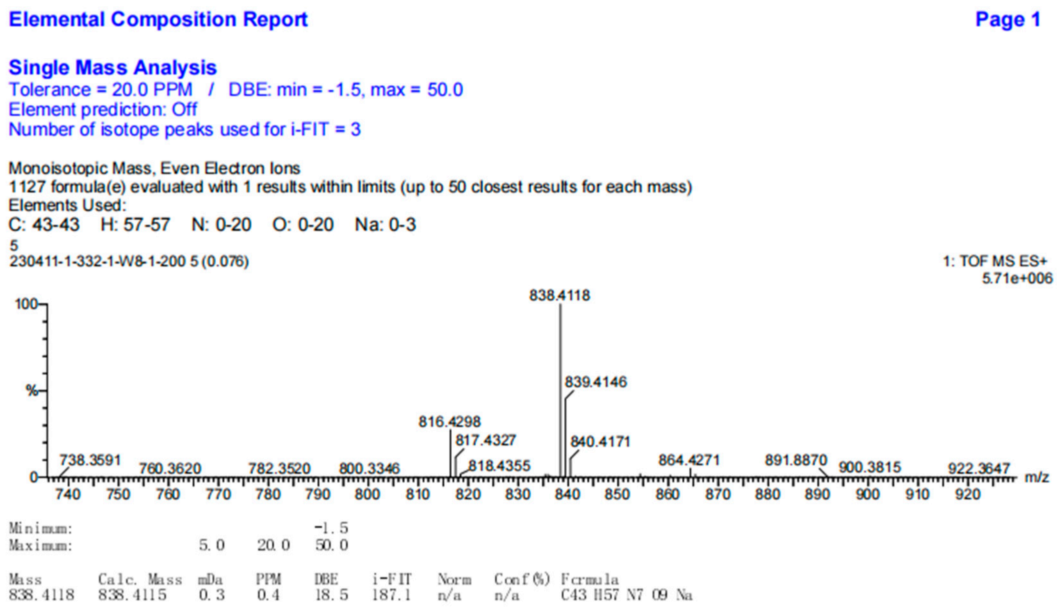

Figure S17: Mass spectrum of compound 17

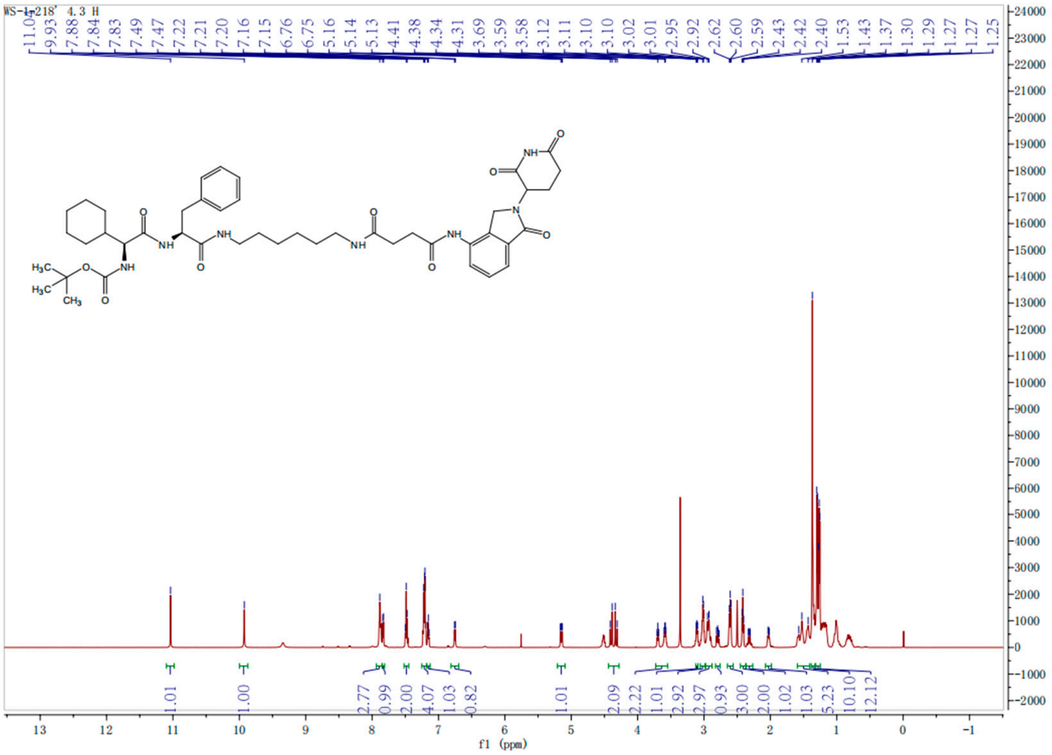

Figure S18: ^{1}H spectrum of compound **18** (600 MHz, DMSO)

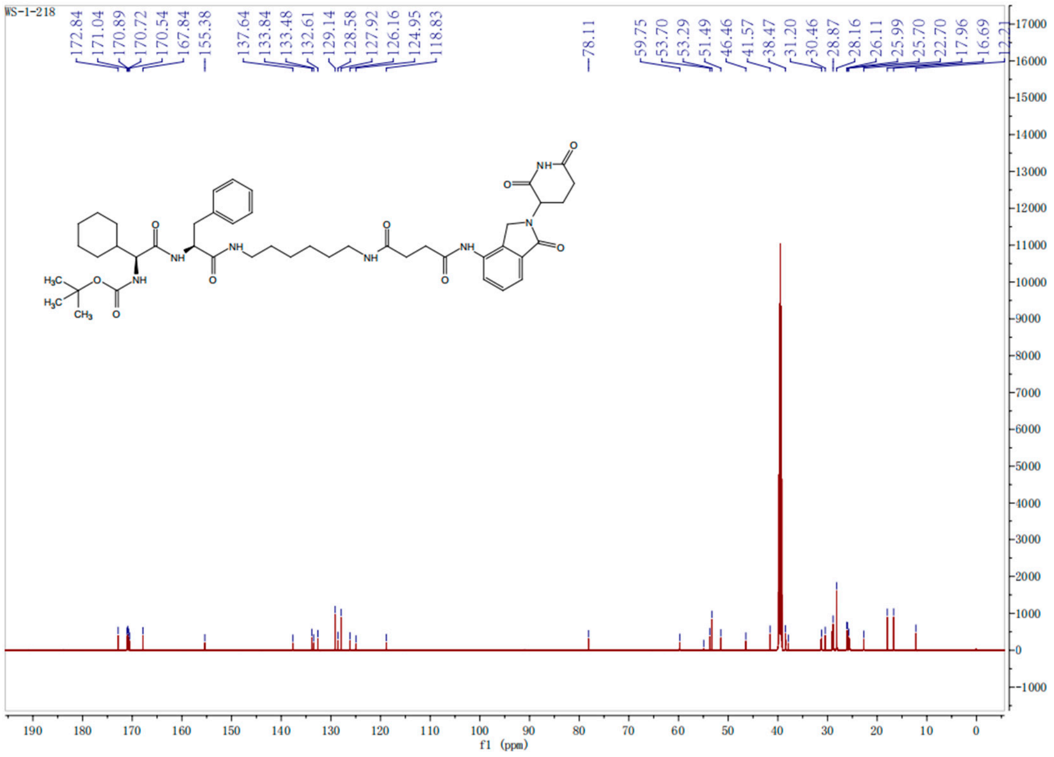

Figure S19:^{13}C spectrum of compound **18** (600 MHz, DMSO)

Elemental Composition Report **Page 1**

Single Mass Analysis
Tolerance = 20.0 PPM / DBE: min = -1.5, max = 50.0
Element prediction: Off
Number of isotope peaks used for i-FIT = 3

Monoisotopic Mass, Even Electron Ions
1097 formula(e) evaluated with 1 results within limits (up to 50 closest results for each mass)
Elements Used:
C: 45-45 H: 61-61 N: 0-20 O: 0-20 Na: 0-3
5
230411-1-332-1-W8-1-218 5 (0.076) 1: TOF MS ES+ 3.52e+006

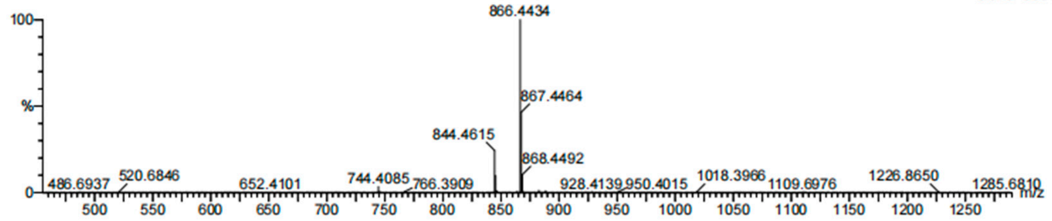

Minimum:				-1.5				
Maximum:		5.0	20.0	50.0				
Mass	Calc. Mass	mDa	PPM	DBE	i-FIT	Norm	Conf(%)	Formula
866.4434	866.4428	0.6	0.7	18.5	171.2	n/a	n/a	C45 H61 N7 O9 Na

Figure S20: Mass spectrum of compound **18**

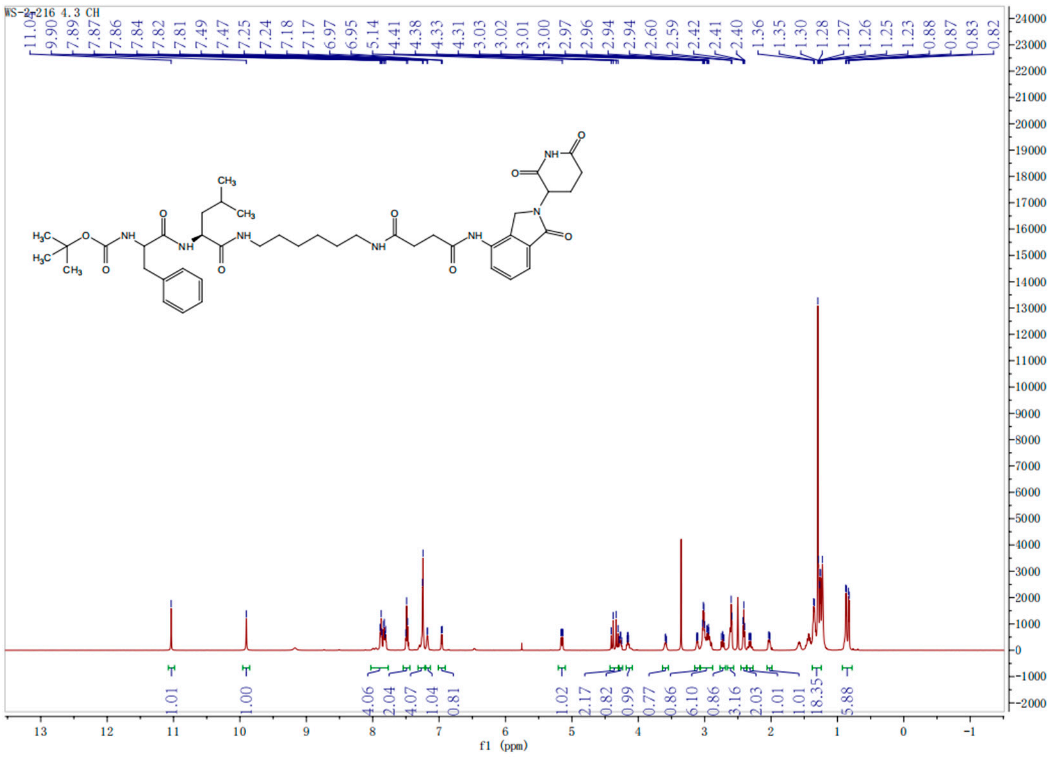

Figure S21: ^{1}H spectrum of compound **19** (600 MHz, DMSO)

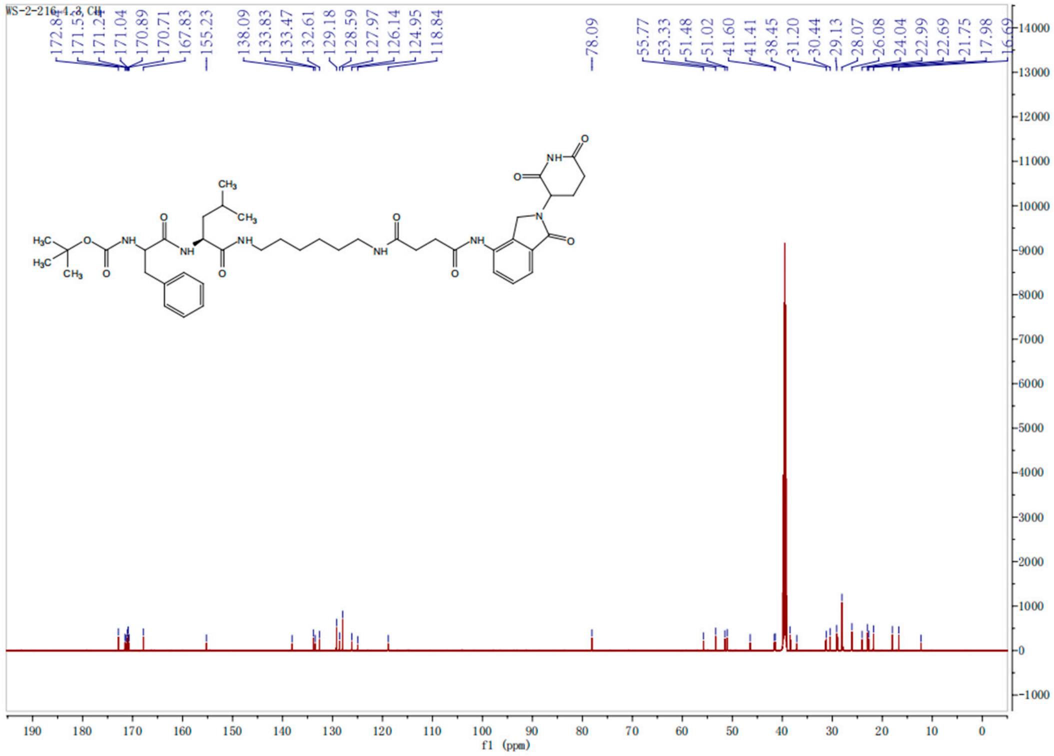

Figure S22:^{13}C spectrum of compound **19** (600 MHz, DMSO)

Elemental Composition Report **Page 1**

Single Mass Analysis
Tolerance = 20.0 PPM / DBE: min = -1.5, max = 50.0
Element prediction: Off
Number of isotope peaks used for i-FIT = 3

Monoisotopic Mass, Even Electron Ions
1117 formula(e) evaluated with 1 results within limits (up to 50 closest results for each mass)
Elements Used:
C: 43-43 H: 59-59 N: 0-20 O: 0-20 Na: 0-3
5
230411-1-332-1-W8-1-216 11 (0.136) 1: TOF MS ES+
8.49e+004

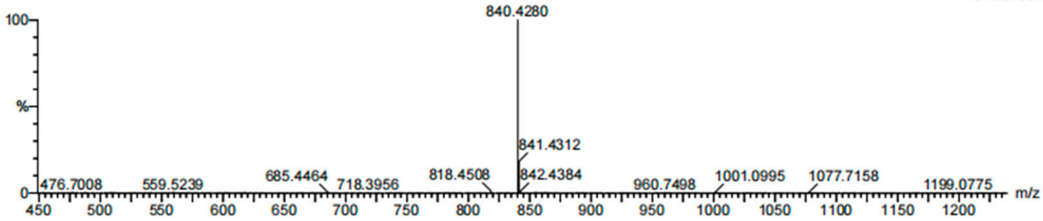

Minimum:				-1.5				
Maximum:		5.0	20.0	50.0				
Mass	Calc. Mass	mDa	PPM	DBE	i-FIT	Norm	Conf(%)	Formula
840.4280	840.4272	0.8	1.0	17.5	100.0	n/a	n/a	C43 H59 N7 O9 Na

Figure S23: Mass spectrum of compound **19**
